# Guideline on diagnostic procedures for suspected hypersensitivity to beta-lactam antibiotics: Guideline of the German Society for Allergology and Clinical Immunology (DGAKI) in collaboration with the German Society of Allergology (AeDA), German Society for Pediatric Allergology and Environmental Medicine (GPA), the German Contact Dermatitis Research Group (DKG), the Austrian Society for Allergology and Immunology (ÖGAI), and the Paul-Ehrlich Society for Chemotherapy (PEG)

**DOI:** 10.5414/ALX02104E

**Published:** 2020-05-28

**Authors:** Gerda Wurpts, Werner Aberer, Heinrich Dickel, Randolf Brehler, Thilo Jakob, Burkhard Kreft, Vera Mahler, Hans F. Merk, Norbert Mülleneisen, Hagen Ott, Wolfgang Pfützner, Stefani Röseler, Franziska Ruëff, Helmut Sitter, Cord Sunderkötter, Axel Trautmann, Regina Treudler, Bettina Wedi, Margitta Worm, Knut Brockow

**Affiliations:** 1 *Clinic for Dermatology and Allergology, Aachen Comprehensive Allergy Center (ACAC), Uniklinik RWTH Aachen, Germany *; 2 *Department of Dermatology, Graz Medical University, Graz, Austria, *; 3 *Department of Dermatology, Venereology and Allergology, St. Josef Hospital, University Hospital of the Ruhr University Bochum, Bochum, *; 4 *Department of Dermatology, University Hospital Münster, Münster, *; 5 *Department of Dermatology and Allergology, University Hospital Gießen und Marburg, Gießen Site, Gießen, *; 6 *Department of Dermatology and Venereology, University, Hospital Halle (Saale), Halle (Saale), *; 7 *Paul-Ehrlich Institute, Langen, *; 8 *Department of Dermatology, University Hospital Erlangen, Erlangen, *; 9 *Asthma and Allergy Centre, Leverkusen, *; 10 *Division of Pediatric Dermatology and Allergology, Auf der Bult Children’s Hospital, Hannover, *; 11 *Department of Dermatology and Allergology, University Hospital Gießen und Marburg, Marburg Site, Marburg, *; 12 *Department of Dermatology and Allergy, University Hospital, LMU Munich, Munich, *; 13 *Institute of Surgical Research, Philipps University Marburg, Marburg, *; 14 *Department of Dermatology and Allergy, Allergy Center Mainfranken, University Hospital Würzburg, Würzburg, *; 15 *Department of Dermatology, Venereology, and Allergology and Leipzig Interdisciplinary Center for Allergology – LICA-CAC, University of Leipzig, Leipzig, *; 16 *Department of Dermatology and Allergy, Comprehensive Allergy Center, Hannover Medical School, Hannover, *; 17 *Department of Dermatology, Venereology, and Allergology, Charité University Hospital Berlin, Allergy Center Charité (ACC), Berlin, and *; 18 *Department of Dermatology and Allergology am Biederstein, School of Medicine, Technical University of Munich, Munich, Germany*

**Keywords:** beta-lactam antibiotics, allergy, penicillin, cephalosporin, drug hypersensitivity

## Abstract

This guideline on diagnostic procedures for suspected beta-lactam antibiotic (BLA) hypersensitivity was written by the German and Austrian professional associations for allergology, and the Paul-Ehrlich Society for Chemotherapy in a consensus procedure according to the criteria of the German Association of Scientific Medical Societies. BLA such as penicillins and cephalosporins represent the drug group that most frequently triggers drug allergies. However, the frequency of reports of suspected allergy in patient histories clearly exceeds the number of confirmed cases. The large number of suspected BLA allergies has a significant impact on, e.g., the quality of treatment received by the individual patient and the costs to society as a whole. Allergies to BLA are based on different immunological mechanisms and often manifest as maculopapular exanthema, as well as anaphylaxis; and there are also a number of less frequent special clinical manifestations of drug allergic reactions. All BLA have a beta-lactam ring. BLA are categorized into different classes: penicillins, cephalosporins, carbapenems, monobactams, and beta-lactamase inhibitors with different chemical structures. Knowledge of possible cross-reactivity is of considerable clinical significance. Whereas allergy to the common beta-lactam ring occurs in only a small percentage of all BLA allergic patients, cross-reactivity due to side chain similarities, such as aminopenicillins and aminocephalosporins, and even methoxyimino cephalosporins, are more common. However, the overall picture is complex and its elucidation may require further research. Diagnostic procedures used in BLA allergy are usually made up of four components: patient history, laboratory diagnostics, skin testing (which is particularly important), and drug provocation testing. The diagnostic approach – even in cases where the need to administer a BLA is acute – is guided by patient history and risk – benefit ratio in the individual case. Here again, further studies are required to extend the present state of knowledge. Performing allergy testing for suspected BLA hypersensitivity is urgently recommended not only in the interests of providing the patient with good medical care, but also due to the immense impact of putative BLA allergies on society as a whole.

First published in Allergo J Int. 2019; 28: 121-151; DOI: 10.1007/s40629-019-0100-8

Development stage: S2k

AWMF guidelines register number: 061-032

Completed: 12. Oktober 2018

Valid until: Oktober 2023

ICD-10 number: Z88.0

## Background 

Beta-lactam antibiotics (BLA) are considered the substance group that most frequently triggers immunologically mediated drug hypersensitivity reactions [1]. [Table Abbreviation]

**Epidemiology:** Approximately 8% of all adults questioned in a southern European survey reported suffering from a drug allergy and 4.5% from an allergy to BLA [2]. A US analysis of patient files reported that as many as 8% of all individuals have a penicillin allergy and 1% a cephalosporin allergy [3]. Around 10% of parents report drug hypersensitivity reactions in their children, 6% report drug allergies, and ~ 3% a BLA allergy. These are confirmed by means of provocation testing in fewer than 10% of children [4]. Likewise, in adults, suspected hypersensitivity was confirmed in only a limited number of cases. This was the case in 7% of patients in a 2010 European study [5] and in less than 2% of patients investigated in a 2013 US study [6, 7].[Table Box1]

All BLA are capable of triggering hypersensitivity reactions. The frequency with which a substance triggers an allergic reaction depends on the substance itself, the frequency with which it is used, and the underlying disease, among other factors. 

The first BLA to be described as a trigger of allergic reactions was benzylpenicillin (BP). It is less frequently used as in the past and has been superseded by aminopenicillins in terms of trigger frequency. Cephalosporins also frequently cause immediate reactions. Likewise, clavulanic acid has recently been reported as a trigger of allergic reactions [1, 8, 9, 10]. 

Impact: The high number of BLA allergies reported in patient histories hampers treating physicians to a significant extent to select an appropriate antibiotic. Patients are often unable to receive the antibiotic of first choice and physicians are forced to resort to broad-spectrum antibiotics in many cases [11, 12, 13]. The treatment costs for patients with a history of BLA allergy are higher than for those of non-allergic patients. The reasons for this include, e.g., the higher cost of broad-spectrum antibiotics and a higher number of hospital days in this patient group [7, 13, 14]. 

Moreover, the greater use of broad-spectrum antibiotics increases bacterial resistance [15], mediated in particular by “collateral damage”. This term refers to the suppression of the normal flora and the selection of antibiotic-resistant microorganisms even at sites far from the actual infection, e.g., in the region of the intestines colonized by bacteria [16]. Thus, patients with an – unconfirmed – history of BLA allergy more frequently exhibited colonization or infection with methicillin-resistant Staphylococcus aureus (MRSA) and vancomycin-resistant Enterococci (VRE); the prevalence of Clostridium difficile was also higher in this patient group [7]. 

This problematic development is underscored by the significant rise in prescribing rates for cephalosporins in recent years [10, 17]. A history of hypersensitivity is given as the reason for prescribing oral cephalosporins instead of penicillin derivatives [10]. 

Moreover, already the suspicion of BLA allergy reduces quality of life in affected patients due to greater anxiety regarding drug treatment [18]. 

A summary of potential effects caused by a history of BLA allergy are the following:

- Limited choice of antibiotics
- More frequent use of broad-spectrum antibiotics at the cost of targeted treatments
- Ineffective treatment of bacterial infection and sub-sequent damage to the patient’s health
- Higher number of sick days and hospital days
- Incorrect assessment of the risk of a BLA allergy. Since BLA allergy is not present in many cases, disregarding a history of allergy often has no consequences. This endangers the health and life of truly allergic patients.
- Lower quality of life among those affected
- Promotion of bacterial resistance
- Higher costs

## Symptoms 

The classification of drug hypersensitivity reactions is based on the temporal course of the reaction, the clinical picture, and the underlying pathomechanism. 

**Temporal classification. **In the national and international literature, an immediate reaction is assumed if the reaction occurs within 60 min [19] or within 6 h [20] of medication use. Immediate reactions mostly occur up to 60 min – and delayed reactions always between over 60 min and up to weeks – following initiation of the triggering medication. Maculopapular exanthema generally manifests between 4 and 14 days following drug initiation. In rare cases, drug reactions with eosinophilia and systemic symptoms (DRESS) occurring up to 8 weeks following medication use have been described ([19] and see also Table 1). 

In the authors’ experience, exanthemas due to BLA can occur (with decreasing probability) 5 – 10 days following discontinuation of the drug. Exanthema occurring after a period of 10 days of BLA discontinuation is, in all likelihood, not caused by BLA treatment. 

**The clinical classification** makes a distinction between immediate and delayed symptoms [19]. 

As with the classification of other anaphylactic reactions [21], the clinical picture of an immediate reaction is subdivided, depending on symptoms, into severity grades I – IV according to Ring and Messmer [22]. In an investigation of patients with immediate BLA allergy (to benzylpenicillins and aminopenicillins), the vast majority of reactions involved more than one organ system, while only urticaria and/or angioedema was seen in a small percentage of patients [23]. 

Delayed (non-immediate) reactions occur in particular following aminopenicillin use and mostly present as MPE [1, 24, 25]. However, special forms as well as severe delayed hypersensitivity reactions also occur. These include acute generalized exanthematous pustulosis (AGEP), DRESS syndrome (or drug-induced hypersensitivity syndrome, DIHS), as well as multiform and sometimes bullous reactions such as Stevens Johnson syndrome (SJS), toxic epidermal necrolysis ( TEN), localized or generalized fixed drug eruptions (FDE), and serum sickness-like symptoms. 

Differentiating between infections and autoimmune diseases can be challenging. 

Parainfectious exanthema in childhood is often misinterpreted as a cutaneous drug reaction. Kawasaki syndrome, a rare but potentially life-threatening differential diagnosis, particularly in infants and young children, also needs to be considered [26, 27, 28]. 

Uncomplicated MPE in children are referred to as “benign rashes” in Anglo-American countries. These do not affect oral or anogenital mucosa, exhibit no blister formation or epidermolysis, are not associated with (atypical) target lesions, do not significantly reduce general condition, and heal spontaneously and completely within a few days [29]. 

They are classified relative to their pathomechanism according to Coombs and Gell. Type I reactions are immunoglobulin E (IgE)-mediated (clinical example: anaphylaxis); type II reactions refer to cytotoxic reactions that can cause hemolytic anemia, agranulocytosis, or thrombocytopenia; type III reactions are immune complex-mediated (serum sickness, hypersensitivity vasculitis); and type IV reactions are T cellmediated. Type IV reactions are further subdivided according to their primary activation of monocytes (type IVa, e.g., allergic contact dermatitis), eosinophil granulocytes (type IVb, e.g., maculopapular exanthema, DRESS), CD4 and CD8 T cells (type IVc, e.g., bullous exanthema), and neutrophil granulocytes (type IVd, e.g., AGEP) [30, 31, 32]. 

## Chemical structure, allergenic determinants, and cross-reactivity 

BLA are subdivided into different classes; see also Supplementary Figure 1; [33, 34]: [Table Box2]

### Chemical structure 

The beta-lactam ring is common to all BLA. In the penicillin group, the beta-lactam ring is connected to a five-member thiazolidine ring and has one side chain (R1). In cephalosporins, the beta-lactam ring is connected to a six-member dihydrothiazine ring; they also have two side chains (R1 and R2). Monobactams have no other ring structures on the beta-lactam ring; only aztreonam is available in this group. Carbapenems, in contrast to penicillin, have a carbon atom instead of sulfur in the thiazolidine ring, which is connected to the beta-lactam ring, as well as side chains at the R1 and R2 position. The clavams have no side chains in the R1 position [25, 35] (see also Supplementary Figure 1). 

### Allergenic determinants 

BLA are haptens that only become immunogenic by binding to a protein structure. Human serum albumin (HSA) is the main carrier protein. Binding to the amino acid, lysine, takes place via opening of the beta-lactam ring. This results in the formation of primarily benzylpenicilloyl (BPO) from benzylpenicillin. For diagnostic purposes, benzylpenicilloyl-octa-L-lysine (BP-OL) or benzylpenicilloyl-poly-L-lysine (PPL) bound via conjugation with octaor poly-L-lysine are used as major determinants [36, 37, 38]. Minor determinants are formed by other bonds. Until recently, these were commercially available for testing in the form of a minor-determinant mixture. For stability reasons, the test preparation currently available contains only one minor determinant (sodium benzylpenicilloate) [39, 40, 41]. 

 Cephalosporin degradation does not follow the same course throughout the group. In many cases, the R2 side chain acts as a “leaving group”; during binding to the carrier protein, the dihydrothiazine ring fractionates while the R2 side chain is eliminated. This leads to increased beta-lactam ring reactivity. Another possible degradation pathway consists in substitution of the R2 side chain. Investigations on specific IgE-inhibition showed that if the R2 side chain and dihydrothiazine residue are lost, molecular recognition is mainly directed to the R1 side chain and the fragment of the beta-lactam ring that binds to the carrier protein [42]. 

### Cross-reactivity 

**Figure 1.**
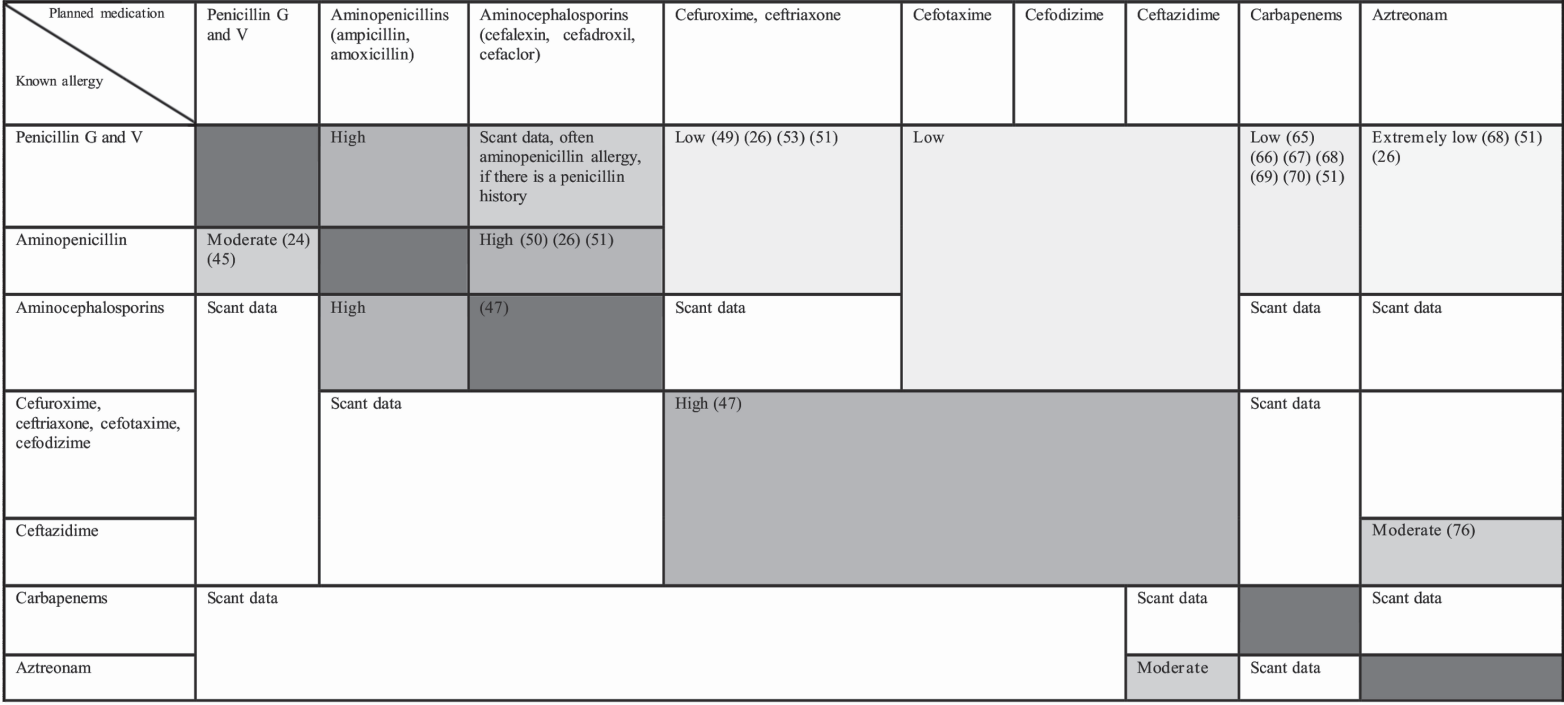
Assumptions on the probability of allergic cross-reactions between the various beta-lactam antibiotics. These assumptions are based on structural similarities or structural differences in the R1 side chain; published data are scant or lacking.

It was originally assumed that the ring structure common to all BLA is the most important allergenic structure. Therefore, it was presumed that allergy to one substance in the BL group meant cross-allergy to all other BLA [35]. However, various allergenic target structures were subsequently identified, producing a far more complex picture of possible cross-reactivity and meaning that the majority of allergic patients were by no means obliged to avoid the entire BLA group (Figure 1). 

Since cross-reactivities are of great interest for clinical routine, information based on studies and findings on structural similarities is provided below, subdivided into the different BLA classes. 

****Cross reactivity between **penicillins******

There is high cross-reactivity between semi-synthetic penicillins with an amino group. The most important allergenic determinant among the aminopenicillins is the R1 side chain. Some allergen-specific antibodies are targeted exclusively against the side chain; sometimes, the ring structure is also required for binding [43]. 

**Immediate reactions:**

- Of those individuals with IgE-mediated allergies to aminopenicillins, some will react selectively to aminopenicillins and tolerate benzylpenicillin, whereas others also react to benzylpenicillin determinants.
- The specificity of the IgE-antibodies closely correlates with the BLA responsible for initial sensitization. IgE from patients that were first sensitized to benzylpenicillin recognizes amoxicillin, whereas IgE from patients initially sensitized to amoxicillin predominantly recognizes amoxicillin and not benzylpenicillin [9].

Thus, a 2001 study on 290 immediate allergic patients found a selective reaction to aminopenicillin in 42.1%, in contrast to 57.9% with non-selective reactions [23]. A recently published study by the same group on immediate reactions to aminopenicillins revealed that only 7/51 (14%) reacted also to BP determinants, consistent with altered prescribing habits [44]. The ratio of benzylpenicillin sensitizations has shifted in recent years in favor of aminopenicillin sensitizations [1]. 

**Delayed reactions:**

- Skin testing in a study published in 2010 on 157 subjects with delayed-type allergy to mainly aminopenicillins demonstrated pure side-chain sensitization in 60% of those investigated; they tested negative to PPL, MDM, and BP [45].

## Cross-reactivity between penicillins and cephalosporins 

Three reaction patterns are known for cephalosporins [46]:

- Selective reactivity for the suspected cephalosporin
- Cross-reactivity with penicillins
- Cross-reactivity with other cephalosporins

High cross-allergenicity between penicillins and cephalosporins was previously assumed on the basis of the common beta-lactam ring; however, this was most likely due to contamination of cephalosporins with benzylpenicillin during the production process used up until the mid-1980s [33, 47]. From today’s perspective, patients that react to the entire group of BLA due to sensitization to the beta-lactam ring are considered isolated cases. For example, only one of 128 patients with previous immediate reactions to penicillins exhibited corresponding sensitization to all BLA tested [48]. 

The cross-reactivities observed between penicillins and cephalosporins are primarily due to similarities in side chains and identical three-dimensional structures [33]. Thus, there is cross-reactivity between aminopenicillins and aminocephalosporins, i.e., cephalosporins with an NH2 group at the R1 position. These include cefaclor, cefalexin, cefadroxil (first-generation oral cephalosporins), and cefatirizine, which is not available in Germany. Cefazolin, which is also a first-generation cephalosporin, does not have an NH2 group. Ampicillin, cefaclor, and cefalexin, as well as amoxicillin, cefadroxil, and cefatirizine, all have the same R1 side chain. 

However, penicillin G and the first-generation cephalosporin, cephalothin, which is not commercially available in Germany, exhibit cross-reactivity, despite different side chains, due to their identical three-dimensional structure [33]. [Table Box3]

**Immediate reactions:**

- In a study by Miranda et al. [49], 38% of the 21 amoxicillin-allergic subjects investigated reacted to cefadroxil.

**Delayed reactions:**

- Clinical studies revealed crossreactivity between (amino-)penicillins and aminocephalosporins in fewer than 40% of subjects.
  – Thus, of the 214 subjects who had experienced a delayed reaction to penicillins (primarily aminopenicillins) in the past, 18.7% tested positive in skin testing with aminopenicillins [25].
  – Another study on 97 delayed allergic patients demonstrated cross-reactivity in 10.9% with the first-generation cephalosporins, cefaclor and cefalexin, in skin tests, whereby cefadroxil was not tested [50].
  – However, it can be said overall that the majority of patients with this type of sensitization to aminopenicillins exhibited no sensitization to aminocephalosporins.

There are also reports in the literature on sensitization among penicillin-allergic individuals to other cephalosporins such as cefoperazone [51], ceftriaxone [51], cefuroxime [50, 52], cefpodoxime, and cefixime [53], as well as to cephalothin and cefamandole [48]. 

**Cefuroxime/cefuroxime axetil.** Cefuroxime and its orally available prodrug, cefuroxime axetil, both of which are very frequently used in Germany, are second-generation cephalosporins that differ – apart from the BL ring – structurally from penicillins. 

**Immediate reactions:**

- A study conducted on 101 penicillin-allergic patients demonstrated no cross-reactivity for IgE- mediated reactions [48].

**Delayed reactions:**

- Of 213 patients with delayed allergy to penicillin, none reacted to cefuroxime axetil [25].
- A study on 97 delayed-type penicillin allergics reported five positive patch test reactions to cefuroxime axetil [50]. [Table Box4]

In the investigation conducted by Caimmi et al., 6.7% of 135 patients with penicillin allergy exhibited sensitization to cefuroxime or cefuroxime axetil in skin tests or a reaction to provocation testing [52]. 

As a prodrug, cefuroxime axetil is converted into cefuroxime only once it has been absorbed by the body. Due to structural differences, false-negative allergy testing for cefuroxime axetil is possible in the case of hypersensitivity to cefuroxime [54]. If a reaction to parenteral administration of cefuroxime is observed, testing with cefuroxime and not solely cefuroxime axetil is recommended. 

**Ceftriaxone.** Ceftriaxone is a third-generation cephalosporin that also differs – apart from the BL ring – structurally from penicillins. The studies that have been carried out were unable to show any crossreactivity with penicillins.

- Immediate reactions: For example, cross reactivity was not observed in any of 101 subjects with immediate allergy to penicillin [48].
- Delayed reactions: Two other studies on 213 and 97 patients, respectively, were unable to verify crossreactivity in delayed allergies.

Essentially, one also needs to talk about cosensitizations and false-positive findings in addition to true cross-sensitivities via the beta-lactam ring. 

Therefore, in summary and contrary to initial reservations, the majority of penicillin-allergic individuals can have access to selected cephalosporins. However, one must not in turn underestimate cross-reactivity, since here too, severe and even fatal reactions have been described [55]. [Table Box5][Table Box6]

## Cross-reactivity of cephalosporins with one another 

Cross-reactivities between cephalosporins occur particularly in the case of similar R1 side chains. 

**Methoxyimino group.** For example, cefuroxime, ceftriaxone, cefotaxime, and cefodizime have a methoxyimino group in the R1 position. The side chains in ceftriaxone and cefotaxime are even identical. Although ceftazidime has a slightly different side chain, it nevertheless sometimes shows cross-reactivity with the above-mentioned substances in patient studies. Of 79 immediate allergic patients that reacted to one active substance in this group, 45.5% tested positive in skin tests to at least one other cephalosporin in the group. If an individual has an immediate reaction to one substance in this subclass, the relative risk of them reacting to another is increased 21-fold, in contrast to individuals that are not allergic to the same substance [46]. 

**Aminocephalosporins.** Another group in which R1 side chain cross-reactivity is seen are the aminocephalosporins, to which cefaclor, cephalexin, cefadroxil, and cefatrizine belong, the latter being unavailable in Germany. Of 15 patients that showed an immediate reaction to cefaclor or cefalexine, four tested positive in skin tests to another aminocephalosporin. The relative risk of a cross-reaction within the group was reported here to be increased 4.46-fold [46]. 

**R2 side chains.** The cephalosporins cefoperazone, cefamandol, and cefotetan, which are not available in Germany, share an identical R2 side chain with a Nmethyl-tetrazole-thiol group. One patient in the study conducted by Romano et al. in 2015 showed crossreactivity between cefoperazone and cefamondole. Cefotetan was not investigated [46, 56]. [Table Box7]

Cephalosporins trigger immediate reactions far more frequently than they do delayed reactions [1]. Also, the suspicion of a cephalosporin as the trigger of a reaction is confirmed more often in immediate than in delayed reactions. For example, suspected delayed allergy to cephalosporins was confirmed in only 5 of 105 patients investigated [57]. In a study on children, none of the assumed cases of delayed cephalosporin allergy could be confirmed, whereas immediate allergy was confirmed in 34 of 43 cases investigated [58]. 

Investigators conducting a study on 105 patients noticed that generalized skin changes in cephalosporin-sensitized patients persisted for 13.6 days on average, in contrast to 3.3 days in non-sensitized subjects [57]. 

**Cross-reactivity between penicillin and carbapenems**

Carbapenems have high structural similarity to penicillins; however, in contrast to penicillins, they do not have a sulfur but rather a carbon atom in the thiazolidine ring. 

Based on an international evaluation of side effects, as well as reported intolerance reactions associated with the use of imipenem/cilastatin, cutaneous hypersensitivity reactions are seen in 2.3 – 2.5% of patients [59, 60]. An incidence of 1.4% is seen for meropenem [61, 62]. 

**Immediate reactions:**

- On the basis of a 1988 study published by Saxon et al., which revealed cross-reactivity of 50% in IgE-mediated reactions, a particularly high reaction rate was assumed. More precisely, in the study by Saxon et al., 10 of 20 patients with a history of immediate reactions who were positive to penicillin or its minor/major determinants also reacted to imipenem or its determinants in skin tests [63].
- However, subsequent studies yielded significantly lower reaction rates to carbapenems (~ 1%) for patients with known immediate allergy to penicillins. For example, in two studies in adults, one of 112 patients was skin test-positive to imipenem/cilastatin [64] and one of 104 patients to meropenem [64]. A pediatric study also demonstrated a positive reaction to meropenem in only one of 107 children [66]. A recently published investigation even revealed tolerability of imipenem/cilastatin, meropenem, and ertapenem in all 211 patients with immediate allergy to penicillins [67]. [Table Box8]

**Delayed reactions:**

- Cross-reactivity was also low in delayed reactions. For example, of 204 patients with known allergy to penicillin none reacted to imipenem/cilastatin or meropenem, as did none of 130 subjects to ertapenem [68].
- In two further investigations by another working group, four of 73 and 97 patients, respectively, with known late reactions to penicillins tested positive to imipenem/cilastatin at patch testing [50, 69].

A retrospective analysis of medical records found that 9.2% of 163 patients with a history of penicillin allergy exhibited hypersensitivity reactions to imipenem/ cilastatin or meropenem, in contrast to 3.9% of the 103 patients with no history of penicillin allergy [70]. 

**Cross-reactivity with monobactams**

Aztreonam is the only monobactam available for clinical use. It is made up of a beta-lactam ring with a side chain and no adjoining ring structure [71]. Aztreonam’s side chain is identical to that of ceftazidime [9]. 

**Immediate reactions:**

- Weak immunogenicity and very low immunological cross-reactivity with BLA (benzylpenicillin and cephalothin) were demonstrated for aztreonam as early on as 1984. Thus, in two investigations, none of 41 and 221 subjects with immediate penicillin allergy, respectively, tested positive [67, 72]. Although two of 29 patients in another study revealed evidence of immediate-type sensitization (skin test or specific IgE), the drug was tolerated in provocation tests [73].

**Delayed reactions:**

- The following studies found no cross-reactivity for delayed reactions. For example, none of 97 patients with known delayed reactions to penicillin or penicillin derivatives tested positive in skin testing, as did none of 76 in drug provocation tests [50]. Likewise, none of 214 patients with known delayed hypersensitivity to aminopenicillins tested positive [25]. [Table Box9]

Heightened caution is warranted with regard to crossreactivity between aztreonam and ceftazidime due to their identical side chains [9]. For instance, a case study reported on a patient with aztreonam and ceftazidime allergy that tolerated benzylpenicillin, amoxicillin, and other cephalosporins [74]. Similarly, a case series of 98 patients with immediate allergy to cephalosporins found cosensitization to aztreonam in 3 patients, with 1 patient showing cross-reactivity between aztreonam and ceftazidime, while 10 other ceftazidime-allergic patients did not develop reactions to aztreonam [75].[Table Box10][Table Box11]

**Beta-lactamase inhibitors**

Clavulanic acid (CLV ) is a BLA that, despite its own weak antibacterial activity but due to its effective inhibition of beta-lactamase, can be used together with amoxicillin (AX). Allergic reactions to clavulanic acid have also been reported [76, 77]. There are no descriptions of cross-reactivity between amoxicillin and clavulanic acid [44]. 

A Spanish investigation on 58 adult patients that had previously experienced immediate reactions to AX/CLV found that 12% reacted to BP determinants, 69% to aminopenicillins while tolerating BP, and 19% to clavulanic acid. Cutaneous testing, as well as drug provocation where indicated, was performed [33, 44]. 

Clavulanic acid sensitization is generally suspected following a reaction to AX/CLV but negative testing to AX and positive skin testing to AX/CLV. However, a study by Torres et al. showed that only 10 of 16 subjects that tested positive to CLV also tested positive to AX/CLV in skin tests, possibly due to the lower test concentration of CLV (4 mg/mL) in AX/CLV compared to 20 mg/mL in the CLV skin test substance [77]. Therefore, testing CLV as a single substance – and not only as a finished medical product together with AX – is recommended following a positive reaction to AX/CLV. The CLV commercially available for skin testing showed a sensitivity of 9 – 18.7% in skin prick tests and 63.6 – 81.2% in intradermal tests [76]. 

## Diagnosis 

### Indication 

Any new reaction in temporal relation to the use of BLA needs to be critically assessed and documented by a physician in a timely manner, where necessary in consultation with an allergist. The unjustified suspicion of BLA hypersensitivity is expressed all too often in routine practice, in spite of the fact that there are more likely differential diagnoses (e.g., infection-related exanthema or acute spontaneous urticaria triggered by infection). The decision on whether hypersensitivity to BLA is likely and requires investigation can only be made on the basis of the clinical picture and the time interval between use of the medication and the onset of the reaction. A (residual) risk assessment is also possible here: in the case of severe clinical manifestations, such as anaphylactic reactions, testing or drug avoidance is necessary in order to protect the patient, even if the likelihood is low. On the other hand, the slightly increased risk of a renewed “benign” exanthema after an incorrectly classified suspected infection-related, uncomplicated maculopapular exanthema, can be taken and justified. As such, patient history and clinical findings must play a key role in terms of establishing the need for testing and planning tests. The precise reconstruction of a reaction years after the event is sometimes challenging for the investigating allergist. [Table Box12][Table Box13]

### Procedure 

Drug allergy testing is more complex than usual allergy testing for protein-based allergens due to the potentially irritative diagnostic methods used and the fact that the majority of allergens are only existing as haptens. Only multifactorial diagnostic methods (skin prick/intradermal tests, serological tests, provocation tests) to complement patient history enable a sufficiently reliable diagnosis of the presence or absence of drug allergy in the majority of cases. 

The reader is also referred to, e.g., the current English-language version of the German guideline for the diagnosis of drug hypersensitivity reactions [19] as well as the recommendations of the European Network on Drug Allergy (ENDA) [26, 78, 79]. Specific issues with particular reference to BLA allergy are highlighted below. 

The diagnostic work-up of drug hypersensitivity comprises four components: patient history, skin tests, in vitro diagnostic methods, and drug provocation tests. The text below is structured according to these components. 

### Patient history 

The patient history is taken from the patients, their parents, or other witnesses. Medical documentation is consulted if possible. 

The correct classification of previous symptoms is crucial to the further approach and the success of subsequent diagnostic procedures. The treating physician determines the further diagnostic work-up on the basis of patient history. 

### In vitro diagnostics 

For in vitro diagnostic procedures, the reader is also referred to both the German and the European guideline on in vitro allergy testing [80, 81]. In vitro testing is of great importance particularly in severe, lifethreatening reactions, since it avoids exposure of the patient to the allergen in question. It enables allergy testing even in high-risk patients, when in vivo testing is contraindicated, and in cases where skin testing is not possible e.g. due to skin disease [26, 81]. 

**In vitro testing for immediate allergies**

- Tryptase (if possible, during the acute reaction and in the further course)
- Specific IgE antibodies
- Cellular in vitro testing

**Tryptase** See also [22, 80]. 

**Diagnostic methods to identify the culprit allergen: quantification of specific IgE. **An immunoassay is used to determine drug-specific IgE (sIgE). A commercial fluorescence enzyme immunoassay (FEIA) is a commonly used test method. There are also other test methods, including an in-house radioimmunoassay or an enzyme immunoassay [9, 81]. A commercially available and valid method for IgE determination is not possible for the majority of BLA; only a determination method **for specific IgE to penicilloyl G and V, ampicilloyl, amoxicilloyl, and cefaclor **is available. 

**Time course.** The level of sIgE to penicillins drops over time if there is no renewed contact with the allergen; however, this occurs to varying degrees depending on the initial level, the type and severity of the reaction experienced as well as on individual factors [82, 83]. 

For example, the elimination half-life (T_1/2_) was 1.6 – 76.4 months in 26 patients investigated. The level remained stable in eight patients over the 55 months measured, while T_1/2_ was less than 6 months in 32% of patients, less than 1 year in 52%, and less than 3 years in 84% [82]. Another study on 41 AX allergic patients made similar findings. It was additionally shown here that negation of a previously positive basophil activation test, which is discussed below, takes place more rapidly than for sIgE. Radioallergosorbent tests (RAST) for the detection of sIgE revealed that 9 patients (22%) were positive at 1 year, four (9.8%) at 2 years, two (4.9%) at 3 years, and 1 patient (2.4%) at 4 years [84]. [Table Box14][Table Box15]

Reversal of previously positive sIgE over time does not mean that the culprit medication will subsequently be tolerated. Thus, reversal was not associated with tolerance upon renewed penicillin use in 63.4% of 22 patients that had experienced penicillinrelated reactions following reversal of a previously positive sIgE to penicillins. Provocation caused a renewed increase in sIgE in some patients [82]. 

**Diagnostic value of specific IgE to BLA. **Study data, particularly on the sensitivity of specific IgE, vary considerably. An important explanation for this lies in the sometimes rapid reversal of positive specific IgE over time. For example, the sensitivity of sIgE to BLA is put at 0 – 75% and its specificity at 66.7 – 100%. The low positive predictive value of 29 – 45.5% is possibly due to cross-reactivity with other allergens; the negative predictive value is 77.1 – 87% [82, 85, 86, 87, 88]. As such, the reliability of specific IgE is the subject of controversy. There are descriptions of patients with clinically relevant sensitization that could not be diagnosed by means of skin testing but only by sIgE, as well as clinical examples in which sIgE yielded no diagnostic information. For example, Torres et al. [89] described 40 of 290 patients that were skin test-negative, but had positive IgE to BLA and clinically relevant sensitization. Macy et al., in contrast, described 4 patients with positive IgE and negative drug provocation tests (DPT), but also six skin test-positive patients with negative sIgE, as well as three that tested positive to DPT but had negative sIgE [90]. 

A study on 171 immediate allergic patients and 122 control subjects showed an improvement in positive predictive value to 92.5% by reducing the threshold value for sIgE to beta-lactams from 0.35 kU/L (kilounit per liter) to 0.1 kU/L, combined with determining a ratio from the sum of BLA-specific IgE and total IgE, which was considered positive at ≥ 0.002. This applied in particular to patients with total IgE of > 200 kU/L [91]. See also Table 2. 

**Diagnostic methods to identify the culprit allergen: cellular diagnosis of immediate allergies.** There are a number of functional assays that can detect cellbound IgE to beta-lactams [92]. Basophils in peripheral blood, on the surface of which allergen-specific IgE antibodies are found, act as effector cells. 

Cellular in vitro tests to diagnose immediate allergy include the:

- Basophil activation test (BAT)
- Cellular antigen stimulation test (CAST, also referred to as CAST-ELISA)
- Histamine release test (HRT)

BAT involves the flow cytometric determination of granulocyte activation markers (CD63 or CD203c) on the surface of basophils as a measure of IgE-dependent stimulation by the drug being tested. CAST and HRT, in contrast, detect mediators that undergo IgE-mediated release. These are sulfo-leukotrienes (CAST) or histamine (HRT). The drugs to be tested are used as liquid allergens. In addition to commercially available solutions, these can also take the form of infusion solutions. This significantly broadens the range of allergens to be tested compared to serological IgE assays [81]. [Table Box16]

**Sensitivity/specificity.** Studies with BLA have shown sensitivities of up to 60% for these three in vitro tests, e.g., 48.6% and 50%, respectively, for BAT [93, 94], 47.7% for CAST [95], and 60% for HFT [96]. Both BAT and CAST showed specificities of over 90% in these investigations, while HRT was much less specific (62.2%). However, due to the heterogeneous patient groups, these findings do not permit direct qualitative comparisons of the tests. Comparative studies were conducted for two of these in vitro assays each. Thus, two investigations found for BAT, in contrast to CAST (and serological IgE diagnostics), sensitivities of 47.8% and 39.1% (BAT), respectively, compared to 41.8% and 22.7% (CAST), respectively, and 30% and 21.7% (sIgE), respectively, and specificities of 83.0% and 93.3% (BAT), respectively, compared to 83.3% and 77.0% (CAST), respectively, and 86.0% and 86.7% (IgE), respectively [97, 98]. Another study compared CAST and HFT in patients with immediate allergy to beta-lactams, with CAST showing a lower sensitivity (43% vs. 53%), but significantly higher specificity (79% vs. 53%) compared to HFT [99]. See also Table 3. 

Possible reasons for false-negative results include: the use of incorrect test concentrations; IgE reactivity to a drug metabolite; non-responders (i.e., failure to activate basophils even in positive controls) in up to 10% of the population [81]; and reversal of a positive test over time following the hypersensitivity reaction. For example, five of 41 patients (12.2%) in one study were still positive for penicillins in BAT after 1 year, 2 patients (4.9%) after 2 years, and 1 patient (2.4%) after 3 and 4 years, respectively [84]. False-positive reactions can occur due to the use of excessively high, non-specifically activating test concentrations or due to cells as yet non-specifically preactivated by the drug reaction [100]. [Table Box17][Table Box18]

**In vitro diagnostics for delayed allergies**

**Cellular diagnostics to identify the culprit allergen.** T-cell assays are primarily used to detect delayed allergies. One should bear in mind here that different mechanisms can underlie the varying clinical manifestations, but that IgE-mediated immediate allergic reactions are also T cell-dependent. In addition, individuals without a history of allergic reactions to BLA may have T-cell clones that react in a specific manner [102]. This means that the results of T-lymphocyte reactions can only be interpreted in conjunction with all other findings and the patient history. 

The following test methods are available following T-cell stimulation by the suspected drug [81, 92]:

- Lymphocyte transformation test (LTT), which determines T-lymphocyte proliferation
- Enzyme-linked immunosorbent spot assay (ELISpot), which determines the number of cells that release relevant cytokines and cytotoxicity markers
- Flow cytometric test methods to determine surface markers and intracellular cytokines
- Enzyme-linked immunosorbent assay (ELISA) to measure released cytokines

**Sensitivity/specificity.** These assays have the greatest significance in the diagnosis of maculopapular exanthema (MPE), fixed drug eruption (FDE), acute generalized exanthematous pustulosis (AGEP), and drug rash with eosinophilia and systemic symptoms (DRESS) or drug-induced hypersensitivity syndrome (DIHS), respectively. 

For example, a number of studies to detect delayed sensitization to BLA in patients with exanthematous reactions found LTT to have sensitivities of between 58% and 68% at high specificities of 91 – 93% [103, 104]. 

As a functional test, the ELISpot assay is possibly more sensitive. For example, a comparative study on amoxicillin allergic patients identified 91% of patients via the detection of interferon gamma (IFN)-γ-producing T cells in the ELISpot (at a specificity of 97%), but only 68% using the LTT (specificity of 85%) [103]. However, since different cytokine patterns may be relevant depending on the patient and the type of reaction (MPE, AGEP, DRESS), several parameters, where possible, such as IFN-γ and interleukin (IL)-5, should be investigated to increase significance [105]. By detecting cytotoxic mediators such as granzyme B (which is also suitable for the detection of exanthematous beta-lactam reactions) or Fas ligand, the ELISpot assay also offers the option to identify a possible trigger, even in severe bullous drug reactions such as erythema multiforme (EM), Stevens-Johnson syndrome (SJS), and toxic epidermal necrolysis (TEN) [106, 107]. 

There are only scant data on the investigation of delayed reactions to beta-lactams in which drugspecific, cytokine-producing T cells have been determined using flow cytometry. A study on 19 patients with different drug reactions, eight of which were triggered by BLA, revealed a sensitivity of 43% each for the cytokines IFN-γ and IL-5, and 79% for both together, at a specificity of 100% [108]. 

**Time of testing.** In terms of the best time for sample collection, there is evidence that performing the LTT for SJS/TEN at the acute stage of disease – more precisely, within 1 week of symptom onset – improved the test’s significance, whereas performing the test for DRESS/DIHS within 5–8 weeks of disease resolution had the highest sensitivity [109, 110]. See also Table 4. [Table Box19]

### Skin tests 

Skin tests are extremely important in the diagnosis of BLA allergies. The classic skin testing methods include the patch test, the skin prick test, and the intradermal test (IDT). The choice of skin test is made on the basis of the suspected pathomechanism of the reaction. These are discussed below. 

The reader is referred to the relevant literature for more details on performing, reading, and evaluating skin tests [19, 26, 78, 79, 111, 112, 113, 114]. 

In contrast to many other drug groups, numerous studies have been conducted on the evaluation of cutaneous allergy testing for BLA. However, this should not obscure the fact that here, too – as in the diagnosis of other drug allergies – numerous issues are the subject of controversy and require further elucidation. 

**Legal basis.** Many BLA are not available as approved test substances for these skin testing methods and need to be manufactured under the direct professional responsibility of the physician for the purpose of personal use in a patient in accordance with § 13 para. 2b of the German Drug Law (*Arzneimittelgesetz*, AMG). The relevant supervisory authorities need to receive one-off notification in accordance with § 67 of the AMG [115]. 

The use of a drug as test material requires the patient’s informed consent. In accordance with § 630e of the German Civil Code (*Bürgerliches Gesetzbuch*, BGB), the physician is obliged to provide the patient with all facts relevant to informed consent. These include in particular the type, scale, performance, expected sequelae, and risks of the procedure, as well as its imperativeness, urgency, suitability, and chances of success with regard to diagnosis or treatment. Documenting the informed consent interview is strongly recommended and the patient should be given a copy of the written patient information and the signed informed consent form. 

**Patch tests**

**Indication.** Patch testing is a method used in the case of suspected delayed reactions. In the case of severe anaphylaxis and suspected high-grade sensitization, open patch testing and 20-min reading can be performed prior to skin prick testing. 

**Time of testing.** Performing patch testing for BLA is recommended 1 month after the skin reaction has resolved at the earliest, but preferably within 1 year of the reaction, since skin test reactivity to BLA diminishes over time [116]. Skin test reactions are altogether rarer in the case of delayed reactions; however, they persist for significantly longer than do immediate reactions [117, 118]. 

**Test substances.** Petrolatum proved to be the optimal vehicle for patch testing with BLA in an investigation conducted using AX and ampicillin (AMP) as examples [114]. In Germany, BLA are used in concentrations of 5 – 10% in petrolatum [78, 112]. The European literature also recommends test concentrations of 10% or 30%; differences have not been reported in petrolatum as yet [114]. Since penicilloyl polylysine fails to yield positive findings in patch testing, it is only used in skin prick and intradermal testing [119]. 

**Variants of classic patch testing.** Since false-negative patch testing may be due to failure of the allergen to penetrate the epidermis, as well as an excessively low test concentration [78], the “strip” patch test [120] and the “scratch-chamber” patch test [121] have become established at some German dermatological departments. 

However, there are no reliable studies as yet for either of these modified patch tests in the diagnosis of BLA allergy, hence only the “classic” patch test is currently recommended in routine allergy practice. 

The “strip” patch test performed according to the standardized protocol can be considered in the case of a negative “classic” patch test but ongoing suspicion of BLA allergy [122, 123]. 

**Skin prick and intradermal tests**

**Procedure. **Skin prick tests should be performed prior to intradermal tests. 

**Time of testing.** Performing skin tests for BLA is recommended 1 month after resolution of the skin reaction at the earliest, but preferably within 1 year of the reaction, since skin test reactivity to BLA diminishes over time [116]. This is particularly important in immediate reactions. 

Background to the recommendation on BLA: Individuals that have experienced immediate reactions may lose their skin test reactivity over time. The longer the time interval between the adverse drug reaction and allergy testing, the greater the likelihood that tests will be negative. For example, of 34 patients with an immediate allergy to a cephalosporin, 62.5% were positive after 1 year, 42.8% after 3 years, and 32% after 5 years [124]. Skin test reactivity remains positive to the culprit drug for longer than other drugs. Individuals that are allergic only to cephalosporins become negative faster and more frequently than do patients that react to penicillins and cephalosporins [124]. Likewise, a faster rate of skin test negativization occurs in selective amoxicillin allergy compared with allergies to benzylpenicilloyl or minor determinants [125]. 

**Test substances for skin prick and intradermal tests**

**Formulation.** If possible, the drug is tested in parenteral form, since this enables intradermal tests with higher sensitivity compared to skin prick tests alone. Test solutions are always freshly prepared [78, 112, 114]. Tablets should be crushed and suspended in saline (0.9% NaCl) for testing; the standardized addition of 1 mL fluid is recommended. Intradermal testing of this preparation is not possible. 

**Minor and major determinants.** It is possible to use benzylpenicillin bound to a transporter protein in skin prick and intradermal tests. The product DAP penicillin® (benzylpenicilloyl-octa-L-lysine as the major determinant and sodium benzylpenilloate as the minor determinant) made by the Spanish manufacturer, Diater, is commercially available for testing in Europe but not approved. It was previously a minordeterminant mix that was reduced to one minor determinant for reasons of stability. The product Prepen® (benzylpenicilloyl polylysine as the major determinant) is distributed on the US market by the company AllerQuest. The studies currently available are on the testing of PPL and MDM – studies for PPL/BP-OL and MD are to follow. 

The value of using minor and major determinants in skin testing for BLA allergies is discussed controversially. The reasons for this include the high cost of commercial substances, problems with the availability of test substances, time-consuming test procedures, as well as regional differences in prescribing habits for BLA and the resulting changes in allergy-relevant allergenic structures. 

A study by Romano et al. on a group of 78 individuals with immediate allergy to penicillins (not aminopenicillins) found that 63 subjects were positive only to PPL and/or MDM and eight only to benzylpenicillin; thus, testing the minor and major determinants was relevant for diagnosis in 81% of these patients [126]. In a study by Bousquet et al., skin testing diagnosed BLA allergy in 136 of 824, while 20 patients tested positive to MDM/PPL only. This means that skin testing with PPL/MDM was required for diagnosis in 14.7% of subjects that tested positive to cutaneous testing, and made drug provocation testing superfluous – or in 2.4% of the total number of patients tested [127]. In a study by Matheu, PPL/MDM testing was required for diagnosis in 47% of 44 skin test-positive patients out of a total of 463 cases investigated [128]. 

**Other test substances.** The remaining BLA are tested cutaneously in unconjugated form. Benzylpenicillin (BP) is used as a complementary test at a concentration of up to 10,000 IU/mL (international unit per milliliter), since this increases test sensitivity compared to testing minor and major determinants alone [26]. 

In addition, amoxicillin and clavulanic acid (DAP® Amoxicillin and DAP® Clavulanic, Diater, Madrid, Spain) are currently commercially available for cutaneous tests. 

It is therefore possible to test the preparation suspected in the past. 

The preparations used (trade names) and their concentrations/potency should be documented. 

**Alternative substances.** The selection of alternative substances to be investigated in the BLA group is based on existing patient findings. It also makes sense to let the selection be guided by what will confer the greatest possible benefit on the patient in the future. 

A possible test series for allergy testing includes:

- For children: benzylpenicillin, phenoxymethylpenicillin, amoxicillin, ampicillin, cefaclor, cefuroxime, and possibly also ceftazidime.
- For adults: benzylpenicillin, phenoxymethylpenicillin, amoxicillin, ampicillin, cefuroxime, cefaclor, cefpodoxime, cefixime, and ceftazidime.

**Test concentrations **(Table 5). Since skin testing can cause severe anaphylaxis in highly sensitized patients [132], titrated testing of the drugs should be performed in high-risk patients and patients with a history of severe drug reactions, starting with a dilution of the maximum test concentration followed by a gradual increase if the result is negative [23]. Open patch testing with a 20-min reading and subsequent initiation of skin prick testing should be considered beforehand. 

**Advantages and disadvantages of intradermal tests in the diagnosis of BLA allergy. **Intradermal tests are more sensitive than skin prick tests in the diagnosis of immediate allergy. The delayed-reading intradermal test is also more sensitive in studies compared to patch testing with BLA [57]. For example, a study on 62 penicillin- and aminopenicillin-allergic individuals found that 4 subjects were only positive in the delayed-reading intradermal test, but not in the patch test [117]. However, only those preparations that are also available in sterile form for parenteral administration can be used in intradermal testing; they cause irritation more frequently and pose a greater risk of anaphylaxis compared to skin prick testing. 

**Diagnostic value of skin prick and intradermal tests in the diagnosis of BLA allergy. **The literature shows a very heterogeneous rate of positive skin tests for immediate reactions to BLA, ranging from 0.8% (4 of 500 [6]) to 73.1% (212 of 290 [67]) and 75.5% (37 of 49 [117]). There is a selection bias here. Patients with high-grade anaphylaxis have positive skin tests more frequently compared to patients with urticaria [67, 133]. There are also differences in test protocols, time intervals between reactions, and the study location, since the frequency in use of different BLA differs between countries. [Table Box20]

The negative predictive value (NPV) of skin testing with PPL and BP or PPL and MDM is 97.74% and 93.02%, respectively. Three out of 130 patients react to DPT following negative skin testing with PPL and BP (2.3%; NPV 97.74). Eight out of 86 patients react following negative skin testing with PPL and MDM (6.97%; NPV 93.02%) [134]. [Table Box21]

Validation of the diagnostic value of testing with the minor determinant (MD) that is now available alone, in contrast to the previously available minor-determinant mix, is pending. The additional benefit of testing with BP, PPL, and MD in relation to the various clinical manifestations of hypersensitivity reactions and the different BLA requires further investigation. 

**Risks of skin tests**

Skin testing with BLA can cause systemic and even life-threatening reactions [132]. These often resemble the original reaction, but are frequently milder [79]. A history of drug-related anaphylaxis is considered a risk factor here. Frequency varies according to patient clientele and test substances, among other factors. For example, a study of 290 patients with immediate allergy to penicillin found that 11% of skin tests caused systemic reactions: 50% to amoxicillin, 29% to BPO, 15% to MDM, and 6% to AMP [23]. Skin testing can also cause a flare-up of delayed reactions; however, there is no evidence that a history of delayed reactions predisposes to anaphylaxis in skin testing. 

Therefore, adequate monitoring is of crucial importance during and after testing [19, 26, 135]. The personnel performing the tests, as well as the infrastructure, need to be prepared for a possible emergency situation. Monitoring for a period of time individually tailored to the patient’s risk, as well as the option to provide immediate emergency care, must be ensured. The individual medical benefit–risk assessment determines whether, where appropriate, allergy skin tests are performed in the inpatient setting. 

The literature reports a higher sensitivity for the detection of penicillin allergies if BP and MD or MDM and PPL/BP-OL are used as complementary skin test substances to the suspected drug. The test substances can be difficult to obtain and are not approved for skin testing. 

Based on an individual risk-benefit assessment, skin testing with the suspected drug as well as BP, MD, and PPL/BP-OL can be useful in the investigation of penicillin allergy, particularly in the case of high-grade anaphylaxis and when caution is required in making the indication for drug provocation testing. 

### Drug provocation testing 

See also [19] for general information on drug provocation testing. 

**Definition.** Drug provocation testing (DPT; also “graded challenge” or “test dosing”, among others) describes the controlled administration of a medication for the purpose of either diagnosing or ruling out a drug hypersensitivity reaction [20]. 

**Background.** Adverse drug reactions can be reproduced independently of their pathomechanism. Patient-specific factors, such as drug metabolization and genetic factors, affect the result. 

Drug provocation testing is the final step in allergy diagnostics, after the patient history has been taken and in vitro methods as well as skin testing have been performed in line with the indication. Particularly in childhood, direct provocation testing is propagated following benign, late onset exanthema [136]. 

For safety reasons, DPT is not performed in the case of a prior positive skin test to the BLA in question and clear patient history [30]. 

Suggestions for dosing steps in DPT can be found in Table 6. 

Test doses need to be calculated for children according to age and weight. 

The product information for the substance in question also needs to be consulted, not least in relation to infusion time, time intervals between administration, and patient-specific factors such as dose adjustment in the case of renal dysfunction. This may mean that medication exposure can take longer. 

 Medical supervision during the follow-up period, including the option to provide prompt intensive medical care, shall be maintained following provocation for as long as severe reactions (e.g., anaphylaxis) can be expected. For this reason, provocation tests likely to cause systemic reactions should be performed in an inpatient setting equipped to provide immediate emergency care (experienced medical and nursing personnel, appropriate drugs and technical equipment). Determining the procedure for drug provocation testing should always remain a case-by-case medical decision that takes numerous individual factors into consideration (e.g., type of drug, estimated likelihood of a reaction, anticipated severity of the reaction, patient expectations/anxiety). [Table Box22]

A normal DPT with BLA has a high negative predictive value. This was 94.1% in a multicenter European study with 1-day provocation. Nine of 118 subjects reported delayed reactions in the follow-up period of at least 6 months; no severe reactions were observed [137]. 

### Desensitization (tolerance induction) 

**Definition.** Drug desensitization (or tolerance induction) describes the triggering of a temporary tolerance to a substance responsible for a drug hypersensitivity reaction [138]. 

Successful desensitization in the case of known immediate allergies to BLA is well documented in the literature. In contrast, there are only a handful of reports on desensitization in mild delayed reactions such as MPE and FDE; success in these cases is the subject of controversy [138, 139]. Desensitization is contraindicated in patients with type II and III reactions according to Coombs and Gell, as well as severe delayed reactions such as SJS/TEN and DRESS/DIHS [138, 139]. [Table Box23]

One must bear in mind that, in contrast to specific immunotherapy, induced tolerance only lasts for hours or days once treatment has been completed [138]. This status can usually be maintained by administering antibiotics at the usual interval of several hours. However, if the antibiotic needs to be administered again following a longer time interval, repeat sensitization is required [140]. 

**Procedure.** Due to the risk of acute allergic reactions during desensitization, the procedure should only be performed under adequate supervision with an intravenous line and monitoring, and assuming that immediate treatment for an acute allergic reaction can be provided [138]. 

The published protocols relate to desensitization in patients with immediate reactions. The initial dose is between 1/1,000,000 and 1/100 of the full therapeutic dose. This dose is determined according to the severity of the index reaction or, in skin test-positive patients, on the basis of skin titration. As such, it may be necessary to modify the desensitization protocol. The last dose administered is generally doubled at the next administration, until the therapeutic dose has been reached. Doses are usually administered every 15 – 20 min [138]. 

Initial doses of 1/1,000,000 and 1/8 are described for desensitization in the case of delayed reactions; the time intervals for dose escalation vary according to the protocols already described and range from 15 min to several days [139]. 

Pretreatment or concomitant administration of antihistamines and glucocorticoids is considered controversial: it carries the risk of suppressing the first signs of anaphylaxis; however, this suppression can make desensitization easier to perform [139]. If hypersensitivity reactions do emerge, drug administration should be ceased immediately and, if necessary, anti-allergy medication administered. The further approach needs to be adjusted to the reaction experienced by the patient. Possible further steps include, e.g., reducing the dose by one or two doses in the protocol, introducing intermediate steps, repeating problematic doses, or also continuing the previous protocol [138, 140]. Treatment should be discontinued in the case of severe events, serum sickness-like symptoms, and blood cell dyscrasias. 

For examples of test protocols: see Table 7, 8, 9. 

### Special aspects in children and adolescents 

BLA are also the group of drugs most frequently associated with drug hypersensitivity reactions in children and adolescents [136]. 

However, hypersensitivity is demonstrated in only a minority of pediatric patients. Thus, studies on selective patient groups recruited mostly in tertiary centers showed that suspected immediate allergic reactions can be confirmed by positive skin tests and/or oral provocation tests in only 0% to a maximum of ~ 31% of cases. In late reactions too, provocation tests confirmed only ~ 7% to a maximum of 16% of the suspected diagnoses [144, 145, 146, 147]. 

As a general rule, and with only a few exceptions, the same diagnostic algorithms apply in children as in adults. Due to the pain associated with cutaneous testing, particularly intradermal tests, it is less well tolerated by infants and small children than by schoolchildren, adolescents, and adults. In addition, other disorders associated with exanthema, ranging from common bacterial and viral infections to extremely rare but potentially life-threatening Kawasaki syndrome, represent important differential diagnoses to BLA-related exanthema [27, 28, 146]. 

Furthermore, it is not uncommon for children taking BLA to develop uncomplicated MPE, also referred to as benign rashes. These show no mucous membrane involvement or blister formation and are associated with mild to moderate pruritus without reducing the patient’s general condition. They usually resolve spontaneously and completely within several days [29]. 

It should also be noted with regard to the BLA group that cefaclor is considered the main trigger of serum sickness-like reactions, which, besides exanthema, can also be associated with arthralgia and persistent fever [136]. 

It should also be emphasized for children that, due to the limited range of alternative antibiotics, BLA is the first-line treatment for numerous diseases. Moreover, the recommendation to avoid BLA for the rest of one’s life without adequate diagnostic confirmation means significantly limiting treatment options for decades due to children’s higher life expectancy. [Table Box24]

### Procedure 

**Figure 2.**
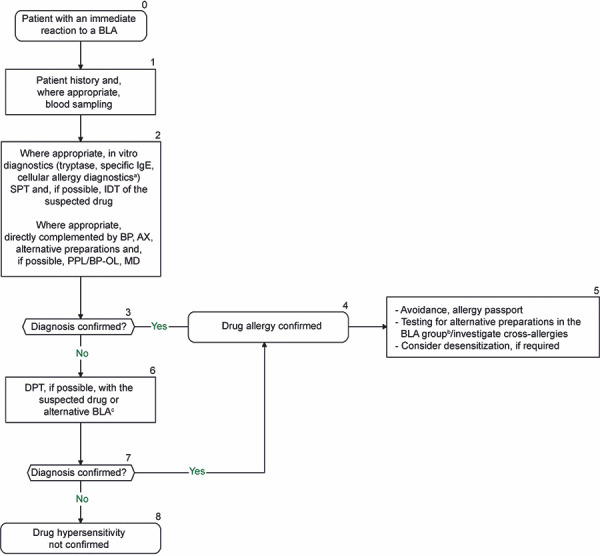
Diagnostic algorithm for immediate reactions to a beta-lactam antibiotic. An individual benefit–risk assessment should be carried out before and after each diagnostic step. ^a^Since positive in vitro testing does not necessarily mean that the positive results are clinically relevant, the physician has the option to decide on a case-by-case basis to continue in vivo testing. However, depending on the individual case, the decision to discontinue further diagnostic steps may also be taken if in vitro testing is positive, either on the basis of sufficiently evaluated evidence of hypersensitivity in the patient history and in vitro testing, or in the case of a negative bene-fit-risk assessment. ^b^Assuming only the suspected BLA has been tested to date: test BP, AX, other alternative preparations, as well as PPL/BP-OL and MD. Alternative preparations need to be determined individually according to the sensitization pattern. A possible test series for alternative preparations in adults includes: BP, phenoxymethylpenicillin, amoxicillin, ampicillin, cefuroxime, cefaclor, cefpodoxime, cefixime, and ceftazidime. For children: BP, phenoxymethylpenicillin, amoxicillin, ampicillin, cefuroxime, cefaclor, and ceftazidime. ^c^If the suspected drug is not tested and administered in DPT, avoidance is recommended and only the BLA tolerated in DPT should be approved. An allergy passport should be issued accordingly. BLA = beta-lactam antibiotic; IDT = intradermal skin test; SPT = skin prick test; BP = benzylpenicillin; AX = amoxicillin; DPT = drug provocation test.

**Figure 3.**
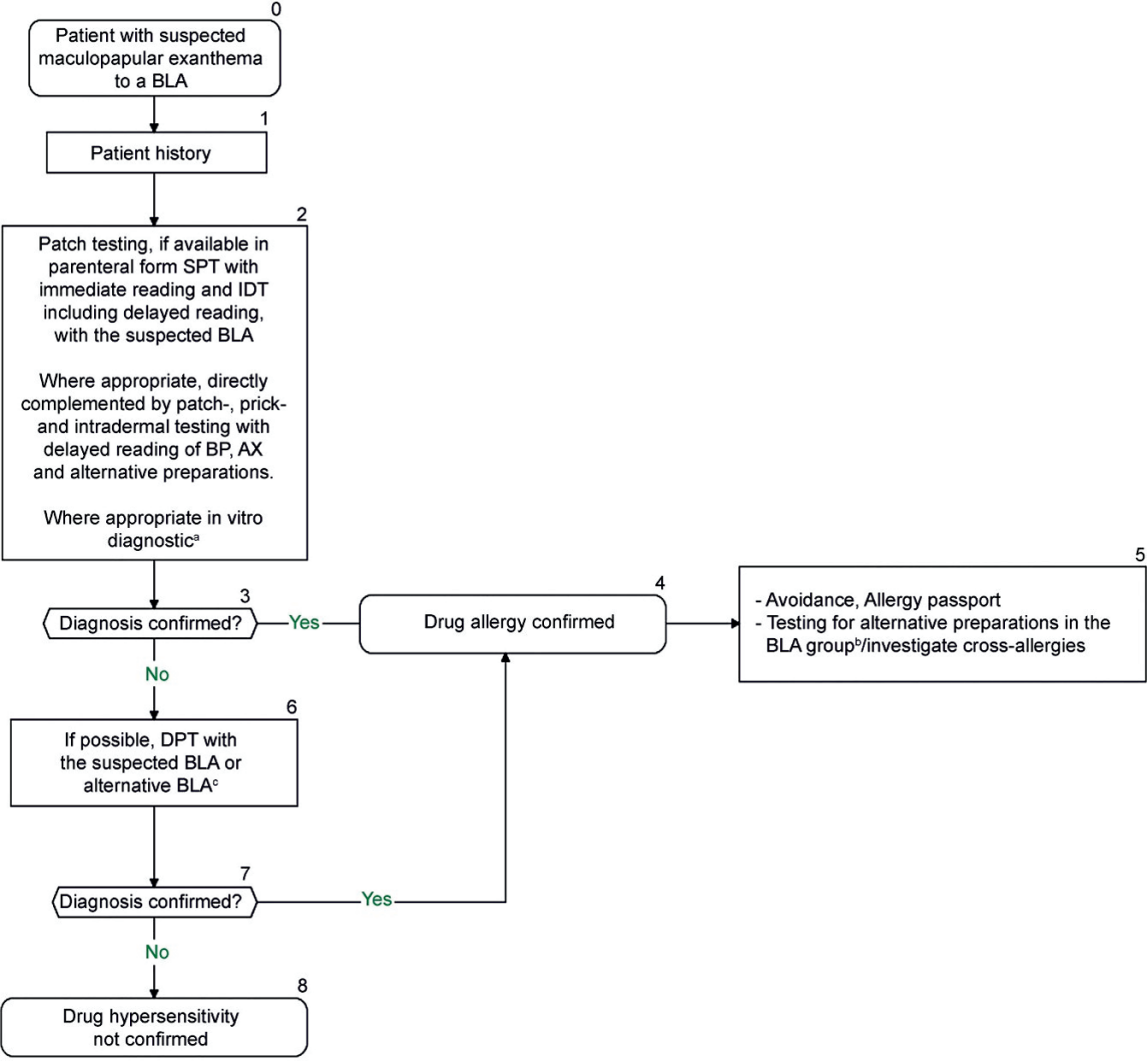
Diagnostic algorithm for suspected maculopapular exanthema to a beta-lactam antibiotic. An individual benefit–risk assessment should be carried out before and after each diagnostic step. ^a^Since positive in vitro testing does not necessarily mean that the positive results are clinically relevant, the physician has the option to decide on a case-by-case basis to continue in vivo testing. However, depending on the individual case, the decision to discontinue further diagnostic steps may also be taken if in vitro testing is positive, either on the basis of sufficiently evaluated evidence of hypersensitivity in the patient history and in vitro testing, or in the case of a negative benefit–risk assessment. ^b^Assuming only the suspected BLA has been tested to date: test BP, AX, other alternative preparations. Alternative preparations need to be determined according to the sensitization pattern. A possible test series for alternative preparations in adults includes: BP, phenoxymethylpenicillin, amoxicillin, ampicillin, cefuroxime, cefaclor, cefpodoxime, cefixime, and ceftazidime. For children: BP, phenoxymethylpenicillin, amoxicillin, ampicillin, cefuroxime, cefaclor, and ceftazidime. ^c^If the suspected drug is not tested and administered as part of DPT, avoidance is recommended and only the BLA tolerated in DPT should be approved. An allergy passport should be issued accordingly. BLA = beta-lactam antibiotic; SPT = skin prick test; IDT = dermal skin testing, BP = benzylpenicillin, AX = amoxicillin; DPT = drug provocation test.

**Figure 4.**
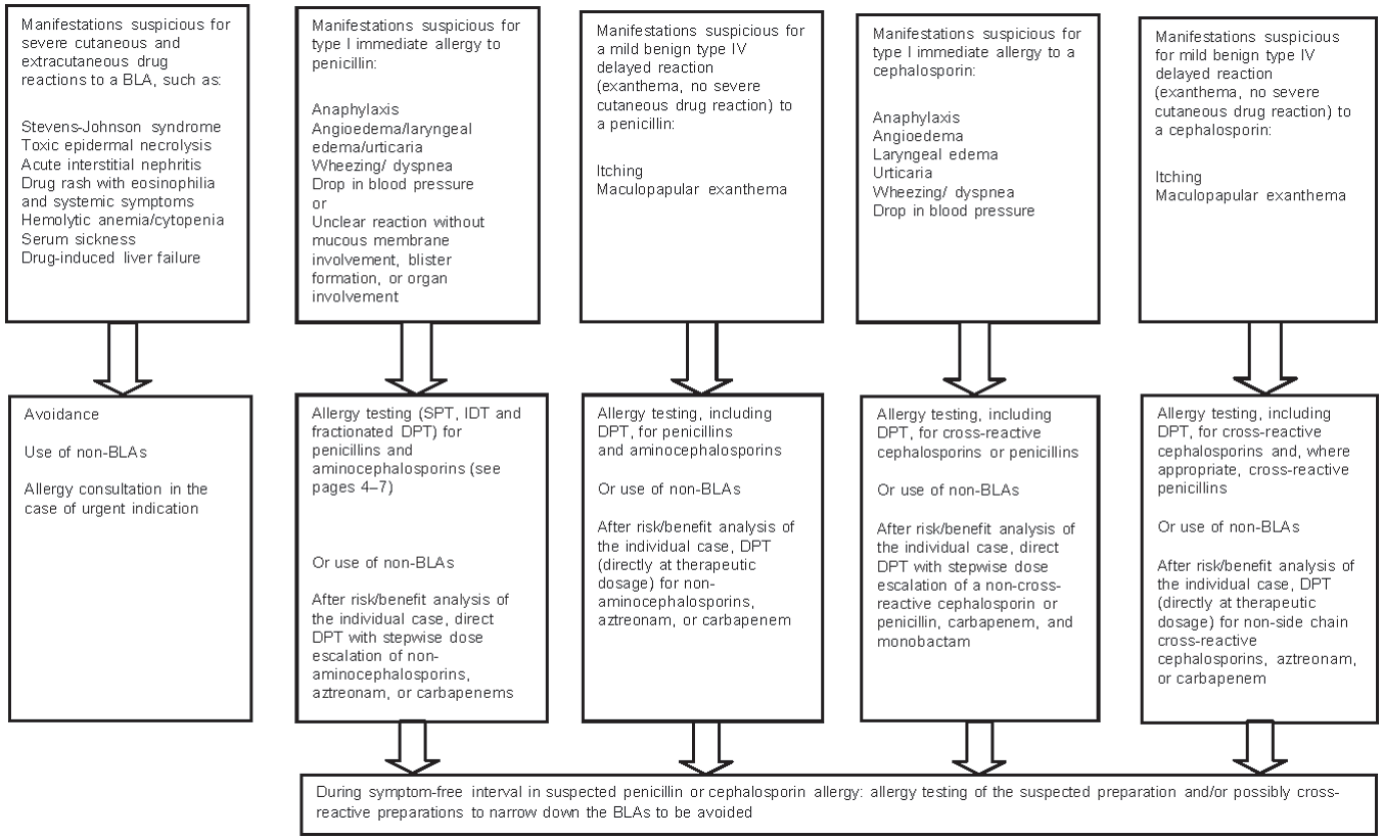
Recommendations for patients with suspected BLA hypersensitivity in cases where treatment is urgently indicated.

The diagnostic procedure is shown in Figure 2, Figure 3. All forms of allergy testing are subject to an individual benefit–risk assessment. Recommendations can be found in Figure 4 on the approach to take if allergy testing cannot be performed in a timely manner in patients with suspected BLA hypersensitivity and an urgent treatment indication. [Table Box25]

**Allergy passport:**

**Figure 5.**
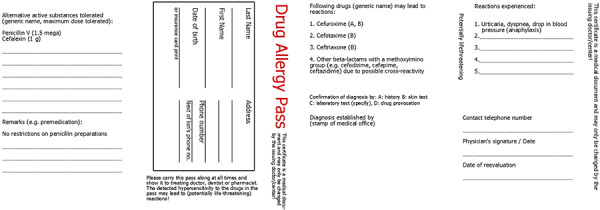
Example of an allergy passport. For a patient with anaphylaxis to cefuroxime who tested positive to other cephalosporins with a methoxyimino group in the R1 side chain (cefotaxime, ceftriaxone) at skin testing, but who exhibited tolerance to the central beta lactam ring structure and an unrelated cephalosporin side chain at skin testing and provocation testing with penicillin V and cefalexin [148].

**Figure 6.**
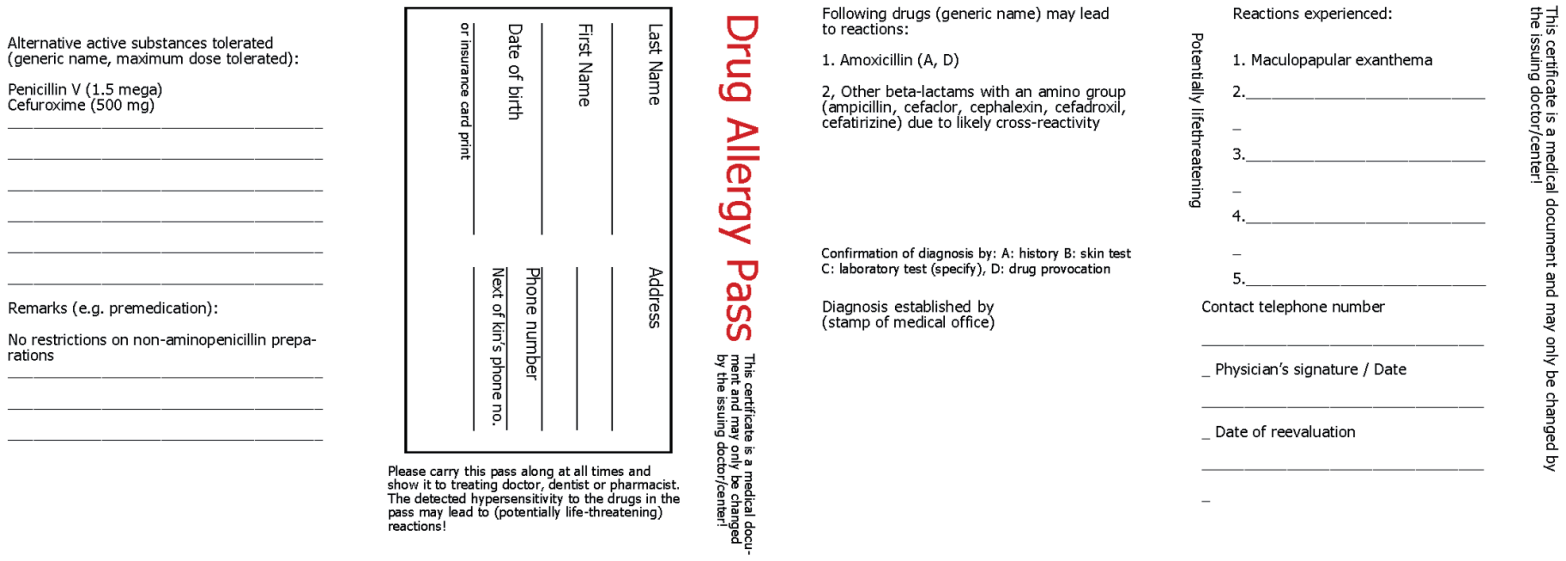
Example of an allergy passport. For a patient with maculopaplar exanthema to amoxicillin for whom beta-lactams with an amino group in the R1 side chain (ampicillin, cefaclor, cephalexin, cefadroxil) were prohibited due to anticipated cross-reactivity, but who exhibited tolerance to the central beta-lactam ring structure and an unrelated cephalosporin side chain at skin testing and provocation testing with penicillin V and cefuroxime [148].

- A document/allergy passport should be issued promptly and indicate hypersensitivity.
- The patient (or parents of affected children) should then be advised on their allergy, the results documented in written form and the patient provided with their results in the form of an allergy passport.
- The allergy passport should be formulated in generally understandable terms and include the clinical presentation of the suspected or diagnostically confirmed BLA allergy, its triggers, as well as drugs and administrable alternative preparations in the BLA group to be avoided in the future.
- If possible, the passport should document the procedure to follow, if a BLA that has not been reliably identified as tolerated urgently needs to be used.
- Generic substance names (INN) and not only trade names should be listed.
- As far as possible, the prohibited substances should be restricted to allergy-relevant drugs.
- Examples are shown in (Figure 5, Figure 6; [148]).

### Research and treatment needs 

Compared to other drug groups, numerous studies have been conducted into the diagnosis of BLA allergies. However, some of the published studies are region-specific in terms of prescribing habits, among other things, but also center-specific in terms of the diagnostic testing performed and the selection of the patient population. Therefore, the sometimes highly heterogeneous results require validation in relation to a variety of factors.

- Clinical data on patient history in relation to test results should be systematically collected and evaluated.
- Cross-reactivities between the BLAs are plausible due to structural similarities, but their clinical relevance requires further investigation.
- The increase in test concentration from 2 to 20 mg/mL for certain cephalosporins in skin prick and intradermal testing requires further investigation, particularly in relation to possible reductions in specificity.
- There are insufficient data on the testing of piperacillin and tazobactam; however they are often used to treat patients. Further investigations are required in order to improve recommendations on testing. Moreover, the allergological relevance of the betalactam inhibitor tazobactam has not been elucidated as yet. Tazobactam is not available for testing as a single substance.
- The usefulness of re-evaluating patients that previously tested negative in allergy testing despite a positive patient history remains unclear due to the highly heterogeneous evidence and requires further investigation.
- The risk of sensitization as a result of allergy testing needs to be investigated further.
- The usefulness of, and risk of sensitization from, the strip patch test with BLA requires further investigation.
- The evidence on which to base diagnostic recommendations in the case of special manifestations is limited, more studies are required.
- In vitro testing has the advantage for the patient that there is no risk of allergic reactions as a result of diagnostic testing. Reliable cellular in vitro testing methods need to be further developed and evaluated.
- The specificity and sensitivity of IgE antibodies to BLA are the subject of controversy; studies are lacking that verify positive results in provocation testing. Specific IgE diagnostic methods are commercially available for only a handful of BLA; more BLA need to be made available.
- Approved test allergens are required for allergy testing.
- The relevance of test preparations such as MD and PPL is the subject of discussion and requires further elucidation.
- All patients with suspected BLA hypersensitivity should undergo allergy testing and the infrastructure for this needs to be created.
- Since allergy testing to BLA is currently not cost-effective, adequate reimbursement for these diagnostic methods is required.

## Funding 

The consensus meetings were financed by DGAKI, the lead organization. Some of the travel expenses for the meetings were financed by the organizations that sent them. No other compensation was paid. 

## Conflict of interest 

Conflict of interest statements – in addition to the guideline report – can be found in tabular form and accessed on the AWMF website for the S2k guideline on “Diagnostics for suspected hypersensitivity to betalactam antibiotics” (www.awmf.org). 

## Open Access 

This article is distributed under the terms of the Creative Commons Attribution 4.0 International License (http://creativecommons.org/licenses/by/4.0/), which permits unrestricted use, distribution, and reproduction in any medium, provided you give appropriate credit to the original author(s) and the source, provide a link to the Creative Commons license, and indicate if changes were made. 

**Table 1.** Typical time intervals between first use of beta-lactam antibiotics and first onset of symptoms (from [20]).

Hypersensitivity reaction	Time interval
Urticaria, asthma, anaphylaxis	Typically up to 1 h, rarely up to 6 h after initial drug administration
Maculopapular drug exanthema	4 – 14 Days after initial drug administration^a^
Fixed drug reaction	1 – 12 Hours after initial drug administration
AGEP	1 – 2 Days after initial drug administration^a^
SJS/TEN	2 – 8 Weeks after initial drug administration^a^

^a^The time interval in renewed reactions is typically shorter compared to initial reactions. In maculopapular drug exanthema, reaction typically after 6 h – 4 days; typical time interval after repeat reactions in AGEP, SJS, TEN, DRESS not investigated . AGEP = acute generalized exanthematous pustulosis; SJS = Stevens-Johnson syndrome; TEN = toxic epidermal necrolysis; DRESS = drug reaction with eosinophilia and systemic symptoms.

**Abbreviations Abbreviation:** Abbreviations

AGEP	Acute generalized exanthematous pustulosis
AMP	Ampicillin
AX	Amoxicillin
BAT	Basophil activation test
BL	Beta-lactams
BLA	Beta-lactam antibiotic/beta-lactam antibiotics
BP	Benzylpenicillin
BPO	Benzyl penicilloyl
BP-OL	Benzylpenicilloyl octa-L-lysine
CAST	Cellular allergen stimulation test
CAST-ELISA	Cellular antigen stimulation test-enzyme linked immunosorbent assay
CLV	Clavulanic acid
DIHS	Drug-induced hypersensitivity syndrome
DPT	Drug provocation test
DRESS	Drug reaction with eosinophilia and systemic symptoms
EM	Erythema multiforme
ELISpot	Enzyme linked immunosorbent spot assay
FDE	Fixed drug eruption
FEIA	Fluorescence enzyme immunoassay
HRT	Histamine release test
HSA	Human serum albumin
IDT	Intradermal test
IgE	Immunglobulin E
IFN-γ	Interferon-gamma
IL	Interleukin
LTT	Lymphocyte transformation test
MD(M)	Minor determinant (mixture)
MPE	Maculopapular exanthema
MRSA	Methicillin-resistant *Staphylococcus aureus*
NORA	Network of severe allergic reactions
NPV	Negative predictive value
PA	Penicillenic acid
PPL	Benzylpenicilloyl-poly-L-Lysin
RAST	Radioallergosorbent test
SDRIFE	Symmetrical drug-related intertriginous and flexural exanthema
sIgE	Specific immunoglobulin E
SJS	Stevens-Johnson-Syndrom
TEN	Toxic epidermal necrolysis
VRE	Vancomycin-resistant enterococci

**Table 2.** Specific IgE.

Advantages	Disadvantages
Testing poses no risk to the patient	Low sensitivity
Serum can be stored and transported	Negativization over time following the reaction
Automated diagnostic testing	Narrow range of allergens

**Table 3.** Basophil activation test.

Advantages	Disadvantages
Testing poses no risk to the patient	Lack of standardization
	Negativization over time following the reaction
Significantly broader range of allergens in contrast to specific IgE	Considerable technical complexity
	Requires fresh blood
	False-negative results or low sensitivity

**Table 4.** LTT/ELISpot assay.

Advantages	Disadvantages
The ELISpot in particular can help to identify the trigger of severe bullous drug reactions in which other test procedures are either not helpful or obsolete	Lack of standardization
This test method yields positive results even years after the event	Technically complex, expensive, and time-consuming
	Requires a large volume of fresh blood
A test method that poses no hazard to the patient	The evidence is insufficient
*ELISpot* enzyme linked immunosorbent spot assay, LTT lymphocyte transformation test	

**Table 5.** A list of test substances and their recommended maximum test concentrations.

Test substance	Maximum skin prick test concentration	Maximum IDT concentration	References
Benzylpenicillin	10,000 IU/mL	10,000 IU/mL	[26]
Amoxicillin	20 mg/mL	20 mg/mL	[114, 116]
Benzylpenicilloyl octa-L-lysine	8.6 × 10^–5^ mol/L	8.6 × 10^–5^ mol/L	[19]
Sodium benzylpenilloate	1.5 × 10^–3^ mol/L	1.5 × 10^–3^ mol/L	[19]
Ampicillin	20 mg/mL	20 mg/mL	[114, 116]
Aztreonam	2 mg/mL	2 mg/mL	[50, 67]
Cephalosporins	2 mg/mL Cefepime 20 mg/mL for cefalexin, cefaclor, cefadroxil, cefuroxime, ceftriaxone, cefotaxime, ceftazidime, cefazolin In the case of a positive reaction, the dose should be reduced by one or two steps of 10	2 mg/mL Cefepime 20 mg/mL for cefalexin, cefadroxil, cefuroxime, ceftriaxone, cefotaxime, ceftazidime, cefazolin	Combined from [25, 130, 131]
Ertapenem	1 mg/mL	1 mg/mL	[67]
Imipenem/cilastatin	0.5 mg/mL	0.5 mg/mL	[64, 67]
Meropenem	1 mg/mL	1 mg/mL	[65, 67]
Piperacillin	20 mg/m	20 mg/mL	[67]

IDT = intradermal test, IU = international units.

**Table 6.** Suggested doses for provocation testing with beta-lactam antibiotics in adultsa.

Active substances	Admin	Therapeutic dose	Commercially available individual quantities [34]	Dose steps; in parentheses, suggestions for low-dose initiation in increased risk of anaphylaxis	Total dose following all dose steps
Benzylpenicillin (penicillin G)	i.v.	1 – 5 million IU/day in 4 – 6 single doses	1, 5, and 10 mega	*(500 IU, 5,000 IU, 50,000 IU)* 500,000 IU, 1,500,000 IU, 5,000,000 IU	7,055,500 IU
Phenoxymethylpenicillin (penicillin V)	Oral	1 – 1.5 mega 3 × daily	1 and 1.5 mega	*(100 IU, 1,000 IU, 10,000 IU)* 100,000 IU, 500,000 IU, 1,500,000 IU	2,111,100 IU
Amoxicillin	Oral	1.5 – 3 g in 3 – 4 SD, increasing to 4 – 6 g	500 and 1,000 mg	*(1 mg, 5 mg, 25 mg) *100 mg, 500 mg, 1,000 mg	1,631 mg
Ampicillin	Oral	2 – 6 g in 3 – 4 SD	500 and 1,000 mg	*(1 mg, 5 mg, 25 mg)* 100 mg, 500 mg, 2,000 mg	2,631 mg
Sultamicillin	Oral	2 × 375 – 750 mg	375 mg	*(0.1 mg, 1 mg, 10 mg)* 37 mg, 187.5 mg, 375 mg	610.6 mg
Flucloxacillin	Oral	1 – 3 g in 1 – 4 SD	500 mg	*(0.1 mg, 1 mg, 10 mg)* 100 mg, 500 mg, 1,000 mg	1,611.1 mg
Piperacillin	i.v.	6 – 12 g, maximum 24 g divided over 2 – 4 SD	1, 2, 3, and 4 g	*(1 mg, 10 mg, 100 mg) *500 mg, 2,000 mg, 6,000 mg	8,611 mg
Mezlocillin	i.v.	3 × daily 2–3 g up to 2 × 10 g	2 and 4 g	*(1 mg, 10 mg, 100 mg) *500 mg, 1,500 mg, 4,000 mg	6,111 mg
Cefaclor	Oral	3 × 500 mg	500 mg	*(0.1 mg, 1 mg, 5 mg) *25 mg, 125 mg, 500 mg	656.1 mg
Cefalexin	Oral	1 – 4 g in 3 – 4 SD	500 mg, 1 g	*(0.1 mg, 1 mg, 10 mg) *100 mg, 250 mg, 1,000 mg	1,361.1 mg
Cefadroxil	Oral	1 – 2× 1g, up to 4 g	1 g or liquid	*(0.1 mg, 1 mg, 10 mg) *100 mg, 250 mg, 1,000 mg	1,361.1 mg
Cefazolin	i.v.	1.5 – 6 g in 2 – 3 SD, depending on pathogen, up to 12 g	1g, 2 g	*(1 mg, 10 mg, 80 mg) *200 mg, 750 mg, 2,000 mg	3,041 mg
Cefuroxime	i.v.	1.5 – 2.25 g in 2 – 4 SD up to maximum 6 g in 4 SD; i.v.: 750 mg or 1.5 g every 8 h	750 – 1,500 mg	*(0.1 mg, 1 mg, 10 mg)* 100 mg, 250 mg, 750 mg	1,111.1 mg
Cefuroxime axetil	Oral	2 × 250 – 500 mg orally	250 mg, 500 mg	*(0.1 mg, 1 mg, 10 mg) *25 mg, 100 mg, 250 mg	386.1 mg
Cefotaxime	i.v.	2–6 g in 2 SD every 12 h	1 g, 2 g	(0.1 mg, 1 mg, 10 mg) 100 mg, 500 mg, 2,000 mg	2,611.1 mg
Cefpodoxime	Oral	2 × 100 – 200 mg, SD every 12 h	100 and 200 mg or liquid	*(0.01 mg, 0.1 mg, 1 mg) *10 mg, 50 mg, 100 mg	161.11 mg
Ceftriaxone	i.v.	1 – 2 g 1 ×/day up to 4 g	500 mg, 1 g	*(1 mg, 5 mg, 25 mg) *100 mg, 500 mg, 1,000 mg	1,631 mg
Ceftazidime	i.v.	2 – 6 g, generally 3 – 4 g	0.5 g, 1 g, 2 g	*(1 mg, 5 mg, 25 mg) *100 mg, 500 mg, 2,000 mg	2,631 mg
Ceftibuten	Oral	400 mg 1 ×/d	200 and 400 mg	*(0.1 mg, 1 mg, 4 mg) *10 mg, 50 mg, 200 mg	265.1 mg
Cefepime	i.v.	2 g every 12 h, maximum every 8 h	1g, 2 g	(1 mg, 10 mg, 50 mg) 100 mg, 500 mg, 2,000 mg	2,661 mg
Imipenem	i.v.	500 mg every 6 h	500 mg (in combination with cilastatin)	(1 mg, 5 mg, 10 mg) 50 mg, 100 mg, 500 mg	666 mg
Meropenem	i.v.	500 mg – 1 g every 8 h, up to 2 g every 8 h	500 mg, 1 g	*(1 mg, 10 mg, 25 mg) *100 mg, 500 mg, 1,000 mg	1,636 mg
Ertapenem	i.v.	1 g 1 ×/day	1 g	*(0.1 mg, 1 mg, 25 mg) *75 mg, 250 mg, 650 mg	1,001.1 mg

^a^The product information for the substance in question also needs to be consulted, not least in relation to restrictions on use, infusion time, time intervals between single doses, and patient-specific factors such as dose adjustment in the case of renal dysfunction. Test doses need to be calculated for children according to age and weight. Time intervals between single doses need to be determined individually; they should be at least 30 min. IU = international units; i.v. = intravenous, SD = single dose, h = hour.

**Table 7.** Combined oral, subcutaneous, and intramuscular penicillin desensitization protocol, administered every 15 min [141].

Number	Units	Mode of administration
1	100	Oral
2	200	Oral
3	400	Oral
4	800	Oral
5	1,600	Oral
6	3,200	Oral
7	6,400	Oral
8	12,800	Oral
9	25,000	Oral
10	50,000	Oral
11	100,000	Oral
12	200,000	Oral
13	400,000	Oral
14	200,000	s.c.
15	400,000	s.c.
16	800,000	s.c.
17	1,000,000	i.m.

s.c. = subcutaneous, i.m. = intramuscular.

**Table 8.** Oral penicillin desensitization protocol, administered every 15 min [138, 142].

Number	Penicillin (mg/mL)	Volumes (mL)	Dose (mg)	Cumulative dose
1	0.5	0.1	0.05	0.05
2	0.5	0.2	0.1	0.15
3	0.5	0.4	0.2	0.35
4	0.5	0.8	0.4	0.75
5	0.5	1.6	0.8	1.55
6	0.5	3.2	1.6	3.15
7	0.5	6.4	3.2	6.35
8	5	1.2	6	12.35
9	5	2.4	12	24.35
10	5	5	25	49.35
11	50	1	50	100
12	50	2	100	200
13	50	4	200	400
14	50	8	400	800

**Table 9.** Intravenous penicillin desensitization protocol using an infusion pump, dose escalation every 15 min [143].

Number	Penicillin (mg/mL)	Flow rate (mL/h)	Dose (mg)	Cumulative dose
1	0.01	6	0.015	0.015
2	0.01	12	0.03	0.045
3	0.01	24	0.06	0.105
4	0.1	50	0.125	0.23
5	0.1	10	0.25	0.48
6	0.1	20	0.5	1
7	0.1	40	1	2
8	0.1	80	2	4
9	0.1	160	4	8
10	10	3	7.5	15
11	10	6	15	30
12	10	12	30	60
13	10	25	62.5	123
14	10	50	125	250
15	10	100	250	500
16	10	200	500	1,000

**Box 1 Box1:** Aim of diagnostic procedures in suspected BLA allergies

The aim of allergy testing is to establish whether a patient with a history of hypersensitivity reaction to BLA actually has an allergy. Knowing that they have a confirmed allergy would protect the allergic patients from further allergic reactions. A prognosis shall be given, which antibiotics not have to be avoided in the future, and the current hypersensitivity shall be investigated.
Qualified allergy testing in patients with a history allergy to one or more BLA makes it possible to select tolerated BLA antibiotics for affected patients in order to more effectively treat bacterial infections. This enables patients to be more frequently treated with the antibiotic of first choice. An infection requiring treatment can be better controlled, resulting in the faster recovery of the patient and fewer infection-related sequelae, not least in terms of patients’ life expectancy.
Targeted treatment of infections reduces the use of broad-spectrum antibiotics, and thus also the selection of resistant bacteria. Antibiotic resistance can be reduced.
The cost to the population as a whole and to the health care system is lowered by the reduction in the use of expensive broad-spectrum antibiotics, fewer sick days and days in hospital, and lower secondary costs resulting from antibiotic resistance.

**Box 2 Box2:** Box 2

- Penicillins
- Benzylpenicillin (penicillin G) and depot forms
- Penicillinase-labile oral penicillins such as phenoxymethylpenicillin (penicillin V)

- Penicillinase-resistant penicillins such as oxacillin, dicloxacillin, and flucloxacillin
- Broad-spectrum penicillins:
- In the aminopenicillin group, such as amoxicillin, ampicillin, and sultamicillin
- Acylaminopenicillins that are also effective against Pseudomonas aeruginosa, such as piperacillin and mezlocillin
- Amidinopenicillins such as pivmecillinam
- Cephalosporins
- Group I: Mainly against gram-positive bacteria, penicillinase-stable, such as the aminocephalosporins cefaclor, cefalexin, cefadroxil, and cefazolin (the latter not belonging to the aminocephalosporins)
- Group II: More effective against gram-negative bacteria, still adequately effective against grampositive bacteria, such as cefuroxime
- Group III: Highly effective in the gram-negative
- range, poor in gram-positive, e.g., cefixime, cefotaxime, cefpodoxime, ceftriaxone, ceftazidime, and ceftibuten
- Group IV: Such as cefepime
- Group IVb respectively V: Against gram-positive and gram-negative pathogens, including efficacy against MRSA, e.g., ceftaroline fosamil, ceftolozane
- Carbapenems such as imipenem, meropenem, and ertapenem
- Monobactams such as aztreonam
- Beta-lactamase inhibitors such as clavulanic acid, sulbactam, and tazobactam.

**Box 3 Box3:** Note

An allergy to all BLA is only present in very few isolated cases.

**Box 4 Box4:** Note

Aminopenicillins cross-react with aminocephalosporins such as cefaclor, cefadroxil, and cefalexin in some patients.

**Box 5 Box5:** Note

Other cephalosporins such as cefuroxime and ceftriaxone show cross-reactivity with penicillins only in individual cases.

**Box 6 Box6:** Note

Cefuroxime, ceftriaxone, cefotaxime, cefodizime, and ceftazidime exhibit possible cross-reactivity due to their side chains.

**Box 7 Box7:** Note

Cefaclor, cephalexin, cefadroxil, and cefatirizine exhibit possible cross-reactivity due to their side chains.

**Box 8 Box8:** Note

Cross-allergenicity between penicillins and carbapenems is low.

**Box 9 Box9:** Note

Cross-allergenicity between penicillins and monobactams is extremely low.
Although ceftazidime and aztreonam have identical side chains, this is of only partial clinical relevance.

**Box 10 Box10:** Recommendations

In the case of patients with a history of immediate reactions to BLA and planned administration of another BLA, skin testing (skin prick test and – if available for parenteral administration – intradermal test) with the planned BLA, in vitro diagnostics where necessary, as well as stepwise drug provocation shall be performed. The range of BLA to be avoided should be kept as narrow as possible.
In the case of patients with a history of immediate reactions to penicillin in whom the use of another BLA is indicated as part of acute emergency treatment and if skin tests are unavailable, fractionated drug provocation tests with a non-aminocephalosporin, aztreonam, or carbapenem under appropriate supervision should be considered after risk/benefit analysis of the individual case. The same applies to the use of a non-side chain-related cephalosporin in patients with a history of immediate reactions to cephalosporins and to the use of aztreonam if there is a history of immediate reactions to all BLA except ceftazidime. Patients with a history of reactions to ceftazidime should only be exposed to aztreonam following negative skin test with the drug.
In the case of a history of immediate reactions or proven allergy to a BLA and urgently indicated use of the suspected BLA or a BLA with a high risk of cross-reactivity, desensitization needs to be considered (see Sect. “Decensitization (tolerance induction)”) after a decision has been taken on the individual case.
In patients with mild delayed reactions (uncomplicated exanthema) to penicillin but urgently requiring another BLA – and allergy testing not possible in a timely manner – the use of a non-aminocephalosporin, carbapenem, or aztreonam is justifiable (albeit associated with an acceptable risk of a similar delayed reaction). The same applies to patients with mild delayed reactions (uncomplicated exanthema) to a cephalosporin in terms of the use of a non-side chain-related cephalosporin, as well as to patients with mild delayed reactions to a BLA other than ceftazidime and the use of aztreonam. If patients have previously reacted to ceftazidime, skin testing should be performed before using aztreonam.
Patients need to be informed about the risk of experiencing similar delayed reactions and instructed on how to respond if a delayed reaction occurs.
If the symptoms of reactions in the patient history cannot be reliably classified (anaphylaxis/urticaria versus uncomplicated exanthema), an approach that assumes prior anaphylaxis shall be selected in the case of an acute need for treatment. It is important when performing allergy testing during a symptom-free interval to establish whether a reaction is immediate or delayed.
In the case of a previous reaction to an aminopenicillin, no aminocephalosporin should be used without prior skin testing. The same approach applies to substances in the side chain-related group: cefuroxime, ceftriaxone, cefotaxime, cefodizime, and ceftazidime with each other.
In the case of previous hypersensitivity reactions to combination preparations containing beta-lactamase inhibitors, hypersensitivity to the beta-lactamase inhibitor is also possible. Therefore, if available, skin testing for this is recommended, as well as provocation testing if necessary.
All recommendations are subject to an individual benefit–risk assessment.

**Box 11 Box11:** Note

Testing clavulanic acid as a single substance for test purposes showed greater sensitivity for the detection of clavulanic acid sensitization compared to testing solely with the finished medicinal product together with amoxicillin.

**Box 12 Box12:** Recommendation

All hypersensitivity reactions suspected of being associated with BLA should undergo diagnostic investigation at any age: on the one hand to identify the trigger and, if possible, the pathomechanism, while on the other, to prevent unnecessary avoidance of BLA by ruling out an allergy. In the case of positive and clinically relevant test findings, possible cross-allergies should be identified or ruled out in order to ensure that patients have access to future BLA treatments. As far as possible, this investigation should be performed within 1 year of the reaction. Prompt diagnosis is particularly important in the case of previous immediate reactions, since test reactivity diminishes over time.

**Box 13 Box13:** Important information when taking a patient history [79].

- Which medications were used prior to and at the time of the reaction (create a timeline if necessary)? Which diseases were already present at that time and were responsible for the use of a BLA?
- Precise chronology:
- The duration of medication use
- The time interval between the last use of the medication and the onset of symptoms
- Duration of the reaction
- Time period to allergy consultation or testing
- Symptoms of the BLA-related reaction (both subjective and objective symptoms) and which organ systems were involved in chronological order of occurrence, as well as laboratory findings and possible treatment interventions.
- Possible augmentation factors, such as infectious diseases and physical exertion, among others.
- Known drug hypersensitivity and other known allergies.
- Previous use and tolerance of BLA.
- General patient history: age, sex, atopy history, other disorders, and current drug use.

**Box 14 Box14:** Recommendations

Serum tryptase determination should be performed within 30–120 min of an acute reaction.
Elevated tryptase during anaphylaxis shall be checked; this shall be performed 24 h after symptoms have ceased at the earliest.
Following severe anaphylaxis in adults, basal serum tryptase shall be determined in order to identify any mast cell diseases.

**Box 15 Box15:** Note

Specific serum IgE diminishes over time in the majority of patients. However, this does not equate to allergen tolerance.

**Box 16 Box16:** Recommendations

Specific IgE determination is recommended within 2 weeks – 6 months following a reaction.
In the case of patients with severe life-threatening reactions, sIgE determination should be performed prior to skin tests and drug provocation tests if possible.
Specific IgE needs to be assessed in the overall context of findings. Since the detection of positive IgE antibodies to beta-lactams is not necessarily of clinical relevance, one can also decide in case of detected specific IgE, in justified cases, to continue in vivo diagnostic testing, including provocation testing to investigate clinical relevance.

**Box 17 Box17:** Note

BAT has the highest significance in the cellular diagnosis of immediate reactions to BLA [101].

**Box 18 Box18:** Recommendations

The cellular diagnosis of immediate reactions can be considered as an optional diagnostic step, in particular prior to skin and provocation testing in high-risk patients, e.g., with a history of high-grade anaphylaxis and if other testing procedures are neither available nor feasible.
Performing the relevant test with different concentrations of the drug to be tested is recommended.
The time window for carrying out cellular diagnosis of immediate reactions should ideally be within 14 days – 6 months following the hypersensitivity reaction.

**Box 19 Box19:** Recommendation

T-cell in vitro assays can be used as an optional complementary testing method for delayed reactions such as MPE, FDE, AGEP, and DRESS if other tests are negative or contraindicated (e.g. in patients following DRESS).
They should be performed 14 days at the earliest following the reaction, but then as soon as possible, even though diagnostically helpful results can still be obtained even after many years.
If possible, T-cell testing for SJS/TEN should be considered within 1 week following symptom onset.
The ELISpot assay can be an instrument to identify the triggering agent in severe drug reactions such as bullous reactions and DRESS/DIHS.

**Box 20 Box20:** Recommendations for skin testing

A skin prick test and (if the preparation is available in parenteral form) an intradermal test are recommended for immediate reactions. Skin prick tests shall always be performed prior to intradermal tests.
In the case of suspected delayed reactions, patch tests and (if the preparation is available in parenteral form) intradermal tests with delayed reading are recommended. Prior to intradermal tests, immediate-reading (and possibly also delayed-reading) skin prick tests should be performed.
In the case of severe delayed reactions, stepwise skin tests should be considered following an individual risk assessment.
If a reaction is equivocal, testing for a possible immediate or delayed reaction is recommended.
Performing skin tests is recommended 1 month after resolution of the skin reaction at the earliest, but preferably within 1 year of the reaction, since skin test reactivity to BLA diminishes over time [116]. This is particularly important in immediate reactions.
After an individual benefit–risk assessment, titrated testing with the medication shall be performed, beginning with a dilution of the maximum non-irritant test concentration, followed by a gradual increase in concentration if the result is negative. Open patch testing with a 20-min reading and subsequent initiation of skin prick testing should be considered.
Testing the suspected drug, if available, on the skin is recommended.
It may be advisable to test CLV as a single substance, if possible, after reactions to AX/CLV.

**Box 21 Box21:** Guidance on the diagnosis in special clinical manifestations.

1. Acute generalized exanthematous pustulosis (AGEP):
- Patch testing shall be performed for diagnostic purposes.
- The reliability and safety of intradermal tests are unclear, while delayed-reading skin prick or intradermal tests can be helpful [116].
- In a French study, 58% of 45 patients tested positive in patch testing following AGEP, but not only in relation to BLA [149], as well as seven of 14 patients in another study [150].
2. Drug rash with eosinophilia and systemic symptoms (DRESS):
- Patch testing should be performed for diagnostic purposes.
- In an evaluation of 14 patients with a history of DRESS and positive patch testing to BLA, and 3 patients with a positive delayed reading after intradermal testing for BLA, none of the patients experienced symptom recurrence [149].
- Diagnostic testing revealed more than one allergen relevant to the previous DRESS in some of the patients (18% in [149]).
- The value of skin prick testing, as well as delayed-reading intradermal tests, remains unclear. Since recurrence has been described, these test methods should only be used in the case of an urgent/vital indication [116].
- T-cell in vitro diagnostics can be an instrument to identify the triggering agent in severe drug reactions such as bullous reactions and DRESS/DIHS.
3. Fixed drug exanthema (FDE):
- Patch testing shall be performed for diagnostic purposes (in loco) [116].
4. Symmetrical drug-related intertriginous and flexural exanthema (SDRIFE)
- Patch testing shall be performed for diagnostic purposes [116].
5. Stevens-Johnson syndrome/toxic epidermal necrolysis (SJS/TEN):
- Patch testing can be considered for diagnostic purposes.
- However, only scant positive results have been described for patch testing in SJS/TEN [78, 116].
- A literature search has not found any evidence as yet that skin testing can cause a renewed flare-up of TEN [112, 149].
- T-cell diagnostics can be considered in individual cases of SJS/TEN.
6. Maculopapular exanthema:
- Patch testing shall be performed for diagnostic purposes. Delayed-reading intradermal testing is recommended if the preparation is available in parenteral form. Before IDT a skin prick test with an immediate-reading should be performed, a delayed reading can be considered.
7. Anaphylaxis and drug-induced urticaria:
- Skin prick testing shall be performed for diagnostic purposes. The IDT test shall be performed for diagnostic purposes if available.
- Specific IgE determination shall be performed for diagnostic purposes if available.
- The basophil activation test can be helpful in some cases.

**Box 22 Box22:** Recommendation

DPT is recommended once other allergy diagnostic tests have been completed, after an individual risk–benefit analysis has been carried out.
If possible, the patient should be exposed to the suspected drug in its original formulation.
In justified exceptional cases, DPT can be performed even without prior diagnostic testing if urgently required for the purposes of administering a drug.
In the case of severe immediate reactions that lie many years in the past and one-off normal provocation testing, a re-evaluation (repetition of skin and in vitro tests, followed by provocation tests if normal) may be considered in individual cases with a high degree of suspicion.

**Box 23 Box23:** Recommendation

Desensitization should be considered as an option if a drug is required in patients with proven or highly likely immediate allergy and no alternative treatment is available or satisfactory. A positive benefit–risk assessment is required.

**Box 24 Box24:** Recommendations for children and adolescents

Allergy testing for a suspected drug hypersensitivity reaction shall be aimed for pediatric patients of all ages.
In the case of a delayed reaction consistent with a benign rash, DPT can be performed without prior cutaneous testing.
However, no DPT should be performed if the delayed reaction is severe.

**Box 25 Box25:** Note

Allergy diagnosis is based on a consideration of all the available information as well as the findings deemed relevant from the patient history, in vitro diagnostics, skin testing, and DPT; the diagnosing physician should also have sound knowledge of the known allergic reactions and allergy-relevant structures.
